# Efficacy and safety of Sihogayonggolmoryeo-tang (Saikokaryukotsuboreito, Chai-Hu-Jia-Long-Gu-Mu-Li-Tang) for post-stroke depression: A systematic review and meta-analysis

**DOI:** 10.1038/s41598-019-51055-6

**Published:** 2019-10-10

**Authors:** Chan-Young Kwon, Boram Lee, Sun-Yong Chung, Jong Woo Kim, Aesook Shin, Ye-yong Choi, Younghee Yun, Jungtae Leem

**Affiliations:** 1 Department of Clinical Korean Medicine, Graduate School, Kyung Hee University, 26 Kyung Hee Dae-ro, Dongdaemun-gu, Seoul 02447 Republic of Korea; 2 Clinical Medicine Division, Korea Institute of Oriental Medicine, 1672 Yuseong-daero, Yuseong-gu, Daejeon 34054 Republic of Korea; 3 Chung-Yeon Central Institute, 64 Sangmujungang-ro, Seo-gu, Gwangju 61949 Republic of Korea; 4 Research and Development Institute, CY Pharma Co., 340, Nonhyeon-ro, Gangnam-gu, Seoul 06227 Republic of Korea; 5 Dongshin Korean Medicine Hospital, 351 Omok-ro, Yangcheon-gu, Seoul 07999 Republic of Korea

**Keywords:** Depression, Stroke

## Abstract

This systematic review and meta-analysis aimed to analyze the efficacy and safety of Sihogayonggolmoryeo-tang (SGYMT), a classical herbal medicine consisting of 11 herbs, for treatment of post-stroke depression (PSD). Thirteen databases were comprehensively searched from their inception dates until July 2019. Only randomized controlled trials (RCTs) using SGYMT as a monotherapy or adjunctive therapy for PSD patients were included. Where appropriate data were available, meta-analysis was performed and presented as risk ratio (RR) or mean difference (MD) with 95% confidence intervals (CIs). We assessed the quality of RCTs using the Cochrane risk of bias tool and the Jadad scale. The quality of evidence for each main outcome was evaluated using the Grading of Recommendations Assessment, Development, and Evaluation (GRADE) approach. Twenty-one RCTs with 1,644 participants were included. In the comparison between the SGYMT and antidepressants groups, the SGYMT group scored significantly lower on both the Hamilton Depression Scale (HAMD) (8 studies; MD −2.08, 95% CI −2.62 to −1.53, *I*^2^ = 34%) and the National Institutes of Health Stroke Scale (NIHSS) (2 studies; MD −0.84, 95% CI −1.40 to −0.29, *I*^2^ = 19%), and significantly higher on the Barthel index (3 studies; MD 4.30, 95% CI 2.04 to 6.57, *I*^2^ = 66%). Moreover, the SGYMT group was associated with significantly fewer adverse events (6 studies; RR 0.13, 95% CI 0.05 to 0.37, *I*^2^ = 0%) than the antidepressants group. In the subgroup analysis, SGYMT treatment consistently reduced HAMD scores within the first 8 weeks of treatment, but thereafter this difference between groups disappeared. Comparisons between SGYMT combined with antidepressants, and antidepressants alone, showed significantly lower scores in the combination group for both HAMD (7 studies; MD = −6.72, 95% CI = −11.42 to −2.01, *I*^2^ = 98%) and NIHSS scores (4 studies; MD −3.03, 95% CI −3.60 to −2.45, *I*^2^ = 87%). In the subgroup analysis, the reductions of HAMD scores in the SGYMT combined with antidepressants group were consistent within 4 weeks of treatment, but disappeared thereafter. The quality of RCTs was generally low and the quality of evidence evaluated by the GRADE approach was rated mostly “Very low” to “Moderate.” The main causes of low quality ratings were the high risk of bias and imprecision of results. Current evidence suggests that SGYMT, used either as a monotherapy or an adjuvant therapy to antidepressants, might have potential benefits for the treatment of PSD, including short-term reduction of depressive symptoms, improvement of neurological symptoms, and few adverse events. However, since the methodological quality of the included studies was generally low and there were no large placebo trials to ensure reliability, it remains difficult to draw definitive conclusions on this topic. Further well-designed RCTs addressing these shortcomings are needed to confirm our results.

## Introduction

Stroke is a common cerebrovascular disease caused by blockage or rupture of the blood vessels responsible for supplying oxygen and nutrients to brain cells. There is growing public interest in this disorder, which is a major global cause of disability and mortality^1^. Among several complications associated with stroke, post-stroke depression (PSD) is one of the most common neuropsychiatric disorders^2,3^, affecting 30–35% of stroke patients^4–8^.

In the Diagnostic and Statistical Manual of Mental Disorders, Fifth Edition (DSM-5), PSD is classified as “*depressive disorder due to another medical condition*^9^”, with five or more major depressive symptoms occurring for 2 weeks or more after the stroke^10^. Several studies have shown that depression is associated with physical disability/recovery and mortality^11–13^. Treatment of PSD is important not only for management of depressive symptoms, but also for improvement of stroke-related treatment outcomes such as the effectiveness of physical and cognitive rehabilitation, and survival rates^2^.

The treatment of PSD is not markedly different from that of depressive disorder and pharmacological treatments using antidepressants, as well as psychotherapy, are frequently recommended^14,15^. Several systematic reviews have supported the efficacy of antidepressants for reducing depressive symptoms^16,17^; but they may not improve the activities of daily living (ADL) of PSD patients, and are more frequently associated with adverse events (AEs) than are placebo treatments^17–19^. Commonly identified AEs, particularly among elderly patients, include an increased risk of falls, hyponatremia, mortality, attempted suicide, and fracture^20,21^. Importantly, the use of antidepressants is also associated with an increased risk of stroke recurrence^22^.

Therefore, the development of a safe and effective alternative treatment for PSD may complement the existing antidepressant-centered strategy, particularly for patients with contraindications to antidepressants. Many aspects of PSD may respond to herbal medicine (HM), one of the modalities of complementary and alternative medicine, because of its multi-compound multi-target nature that potentially benefits neurological function, rehabilitation outcome, quality of life, and depressive symptoms^23^. Sihogayonggolmoryeo-tang (SGYMT, also known as Saiko-ka-ryukotsu-borei-to), is a HM consisting of 11 herbs. It was first introduced in the classical Chinese text “Treatise on Cold Damage Diseases” in the 3rd century. This prescription has since been recommended for several neuropsychiatric conditions including psychological anxiety, neurosis, and irritability^24^. In a recent meta-analysis of 8 randomized controlled trials (RCTs), SGYMT used as a monotherapy or adjunctive therapy to antidepressants was more effective for treating depression than antidepressants alone^25^. Moreover, experimental studies reported that SGYMT had an antidepressant effect by prevention of prefrontal cortex dysfunction^26^, and was as effective as the tricyclic antidepressant (TCA) imipramine^27^. In addition, an RCT reported that 3 months of SGYMT administration modulated dyslipidemia, a risk factor for ischemic stroke, suggesting a positive indirect effect on stroke-related outcomes^28^. Importantly, the use of SGYMT for treating PSD was recommended in a recent traditional Korean medicine (TKM) clinical practice guideline (CPG) in Korea^24^.

However, the use of SGYMT, which may complement the limitations of conventional therapies for PSD, has not yet been systematically and critically reviewed. The objective of this review is to analyze the effectiveness and safety of SGYMT as monotherapy or adjunctive therapy in patients with PSD using a systematic review methodology to help clinicians establish evidence-based treatment strategies for this disorder.

## Materials and Methods

This systematic review was conducted according to the guidelines in the Cochrane Handbook for Systematic Reviews of Interventions^29^. We reported the review according to the Preferred Reporting Items for Systematic Reviews and Meta-Analyses guidelines^30^. The protocol was published^31^ and registered in the PROSPERO (registration number, CRD42018102939).

### Data sources and search strategy

This method was carried out as described previously^31^. Two researchers (CY Kwon and B Lee) performed independent comprehensive searches of the following 13 databases: 6 English-language databases (MEDLINE via PubMed, EMBASE via Elsevier, the Cochrane Central Register of Controlled Trials [CENTRAL], the Allied and Complementary Medicine Database [AMED] via EBSCO, the Cumulative Index to Nursing and Allied Health Literature [CINAHL] via EBSCO, and PsycARTICLES via ProQuest), 5 Korean-language databases (Oriental Medicine Advanced Searching Integrated System [OASIS], Koreanstudies Information Service System [KISS], Research Information Service System [RISS], Korean Medical Database [KMbase], and Korea Citation Index [KCI]), and 2 Chinese-language databases (China National Knowledge Infrastructure [CNKI] and Wanfang Data). The initial search date was July 31, 2018 and we conducted another search for updated information on July 2, 2019 to provide more up-to-date and comprehensive evidence. We identified additional trials using the reference lists of relevant papers and a manual search on Google Scholar. In addition to peer-reviewed articles in scientific journals, we included grey literature such as degree theses and conference proceedings. There was no restriction on language. The following search terms were used in MEDLINE: (“depressive disorder” [MeSH Terms] OR “depression” [MeSH Terms] OR depressive OR depression) AND (“stroke” [MeSH Terms] OR stroke) AND (Chai-Hu-Jia-Long-Gu-Mu-Li-Tang OR Chai-Hu-Jia-Long-Gu-Mu-Li-Wan OR Chai-Hu-Jia-Long-Gu-Mu-Li-Pian OR Saikokaryukotsuboreitou OR Saikokaryukotsuborito OR Sihogayonggolmoryeo-tang) (Supplemental Digital Content 1, which describes the details of search terms used in all databases.

### Inclusion criteria

#### Types of studies

This method was carried out as described previously^31^. We included only RCTs, and excluded quasi-RCTs using inappropriate random sequence generation methods. Studies using the expression “randomization” (随机) without descriptions of randomization methods were included. We included both parallel and crossover studies. In crossover designs, only first-phase data were used to calculate the effect size and in the meta-analysis. Other designs such as *in vivo*, *in vitro*, case reports, retrospective studies, and non-randomized controlled trials were excluded.

#### Participant characteristics

This method was carried out as described previously^31^. We included studies on patients diagnosed with depression following stroke using standardized diagnostic tools such as the DSM-5, regardless of sex, age, or race. Studies were excluded if the participants had drug allergies or other serious illnesses such as cancer, liver disease, or kidney disease.

#### Intervention types

This method was carried out as described previously^31^. We included studies using SGYMT, i.e. 11 kinds of herbs including *Bupleuri Radix*, *Pinelliae Rhizoma*, *Ramulus Cinnamomi*, *Poria*, *Scutellariae Radix*, *Jujubae Fructus*, *Ginseng Radix* or *Codonopsis Radix*, *Ostreae Concha*, *Fossilia Ossis Mastodi*, *Zingiberis Rhizoma Recens*, and *Rhei Rhizoma*. Given that HMs, such as SGYMT, are also known as so-called “modified HM,” which allow some modifications of their compositions to achieve increased efficacy^32–34^, we also included studies using modified SGYMT, which was defined in this review as SGYMT containing more than 50% of the original prescription composition (i.e. HM designated as “modified SGYMT”, which contained 6 or more of the 11 basic components). We allowed the use of any form of SGYMT. Studies combining SGYMT with other therapies as treatment interventions were included, if the other therapies were used equally in both the treatment and control groups. For the control intervention, we included studies that used placebos, no treatment, and conventional medical treatments. We excluded studies using HM as the control intervention because these studies could not yield the net effect of SGYMT. There were no other restrictions regarding the control intervention.

#### Outcome measures

This method was carried out as described previously^31^. The primary outcome measures were (1) post-treatment value in the degree of depression measured by the Hamilton Depression Scale (HAMD)^35^ or Beck Depression Inventory (BDI)^36^ and (2) AEs measured by the Treatment Emergent Symptom Scale (TESS)^37^ or the incidence. The secondary outcome measures included total effective rate (TER), a non-validated outcome measure that is processed secondarily according to certain evaluation criteria such as clinical symptom improvement, or the improvement rates of other quantified outcomes. In the assessment of TER, participants are generally classified as “cured”, “markedly improved”, “improved”, or “non-responder” after treatment. TER is calculated consistently using the following formula: *TER* = *N1* + *N*2 + *N3*/*N*, where *N1*, *N*2, *N3*, and *N* are the number of patients who are cured, markedly improved, improved, and the total sample size, respectively. We also evaluated post-treatment value in neurological function by the National Institutes of Health Stroke Scale (NIHSS), a tool used to quantify stroke-related impairment^38^, measured ADL by the Barthel index, a tool used to describe ADL and mobility^39^, and measured the quality of life by the 36-Item Short Form Health Survey, a patient-reported survey of their own health^40^ as secondary outcome measures.

### Study selection

After removing duplicates, two researchers (CY Kwon and B Lee) independently screened the titles and abstracts of all searched studies for relevance and then evaluated the full texts of the eligible studies for final inclusion. Any disagreement about study selection was resolved through discussion with other researchers, as previously reported^31^.

### Data extraction

This method was carried out as described previously^31^. Two researchers (CY Kwon and B Lee) independently performed and crosschecked the data extraction using a standardized data collection form (Excel 2007, Microsoft, Redmond, WA, USA). Discrepancies were resolved through discussion with other researchers. The extracted items included the first author’s name; year of publication; country; sample size and number of dropouts; details about the participants, HM, control intervention, and comparisons; duration of the intervention; outcome measures; and AEs associated with interventions. We contacted the corresponding authors of the included studies by e-mail to request additional information if the data were insufficient or ambiguous.

### Quality assessment

This method was carried out as described previously^31^. Two researchers (CY Kwon and B Lee) independently assessed the methodological quality of all included studies, and the quality of evidence for each main finding. We resolved discrepancies through discussion with other researchers.

The methodological quality of the included studies was evaluated using both the Cochrane Collaboration’s risk of bias tool^41^ and the Jadad scale^42^. Using the Cochrane risk of bias tool, the following domains were assessed: random sequence generation, allocation concealment, blinding of participants and personnel, blinding of outcome assessments, incomplete outcome data, selective reporting, and other potential biases for each included study. Each domain was categorized into one of three groups: “low risk,” “unclear,” or “high risk.” In the random sequence generation domain, we assessed a study as high risk of bias if the expression “randomization” was mentioned without a description of randomization methods. We assessed other potential sources of bias with particular emphasis on possible baseline imbalances arising from *a priori* selection characteristics for treatment and control groups, such as mean participant age, or baseline depression level. Baseline imbalance arising from selection characteristics that are strongly related to outcome measures may bias the estimation of intervention effects in RCTs^41^. When using the Jadad scale, randomization method, blinding, and descriptions of withdrawals and dropouts are assessed, and the total score is presented on a scale of 1–5.

The quality of evidence for each main outcome was evaluated by using the Grading of Recommendations Assessment, Development, and Evaluation (GRADE) approach^43^. Using the online program GRADEpro (https://gradepro.org/), we assessed the risk of bias; inconsistency, indirectness, and imprecision of the results; and the probability of publication bias using a four-item scale (“Very low”, “Low”, “Moderate”, or “High”).

### Data synthesis and analysis

This method was carried out as described previously^31^. We used Review Manager version 5.3 software (Cochrane, London, UK) for data synthesis and analysis. Descriptive analyses of details of the participants, interventions, and outcomes were conducted for all included studies. Meta-analysis was performed for studies using the same types of intervention, comparison, and outcome measure. We pooled continuous outcomes as the mean difference (MD) with 95% confidence intervals (CIs), and dichotomous outcomes as a risk ratio (RR) with 95% CIs. Heterogeneity of effect measures between studies was assessed using both the chi-squared test and the I-squared statistic (*I* ^2^). We considered *I* ^2^ values greater than 50% and 75% indicative of substantial and high heterogeneity, respectively. In the meta-analyses, a random-effects model was used when the heterogeneity was significant (*I* ^2^ > 75%), while a fixed-effects model was used when the heterogeneity was non-significant. We planned to do this; however, during the review process we learned that this practice was no longer supported and that a random-effects model was preferable because of given potential heterogeneity in true treatment effects due to differences in the treatment components, research groups, and patient selection criteria among the included studies. Therefore, we reported both the results of the models that were pre-registered and those of potentially more appropriate random-effects models. However, we used only fixed-effects models when the number of studies included in the meta-analysis was less than 5, in which the estimates of between-study variance had poor accuracy^44,45^. If the necessary data were available, we conducted a subgroup analysis to account for the heterogeneity or to assess whether the treatment effects vary between subgroups according to the following criteria: (1) the treatment period; (2) the dosage form of SGYMT, such as decoctions or granules; (3) the presence or absence of a placebo; (4) the severity of depression; and (5) the types of antidepressants used. In addition, we performed sensitivity analyses to identify the robustness of meta-analysis results by excluding (1) studies with high risks of bias (2), studies with missing data, and (3) outliers that are numerically distant from the rest of the data. If more than 10 trials were included in the meta-analysis, reporting biases such as publication bias were assessed using funnel plots. When reporting bias was implied by funnel plot asymmetry, we attempted to explain possible reasons for this. Additionally, we used Egger’s linear regression analysis and Begg and Mazumdar’s rank correlation analysis to assess publication bias with Stata/MP version 15.1 software^46,47^.

## Results

### Description of included studies

**Figure 1:**
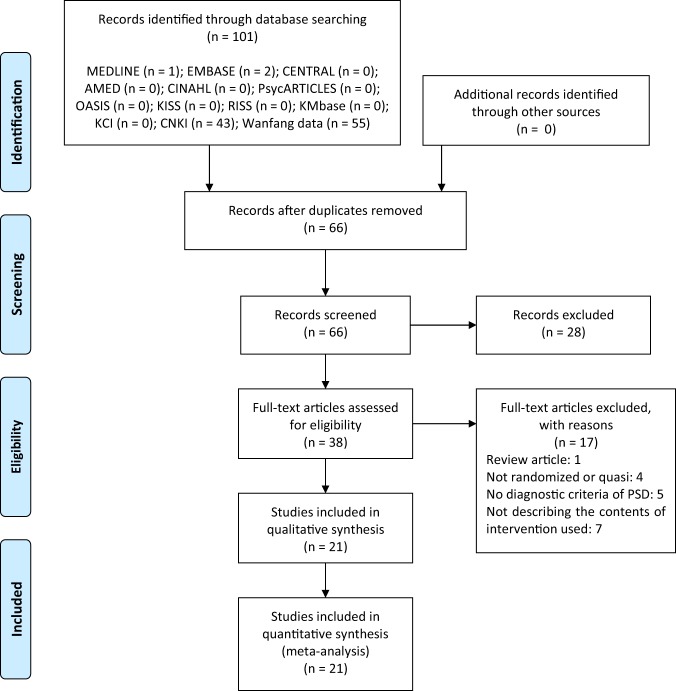
PRISMA flow chart of the study selection process. Moher, D. *et al*. Preferred reporting items for systematic reviews and meta-analyses: the PRISMA statement. PLoS Med 2009; 6(7)e1000097.

We identified a total of 101 records through database searching. After screening of titles and abstracts, 38 articles were considered to be relevant. Among them, 1 review article, 4 non-RCTs or quasi-RCTs, 5 not describing the diagnostic criteria of PSD, and 7 not describing the contents of conventional medication prescribed were excluded by reviewing the full-texts. In total, 21 RCTs with 1,644 participants were included in this review and meta-analysis (Fig. 1)^48–68^.

The general characteristics of the included studies are summarized in Table 1. All RCTs were conducted in China. One was a thesis^50^, 1 was a conference proceedings^48^, and the remaining 19 were journal articles. Thirteen RCTs compared SGYMT to antidepressants^48–60^, and the other 8 compared SGYMT combined with antidepressants to antidepressants alone^61–68^. We were unable to find any placebo-controlled trials. Sample sizes ranged from 48 to 165 with a median of 70, and treatment periods ranged from 14 to 90 days with a median of 42 days. Five studies^48,52,59,60,64^ recruited participants with specific traditional Chinese medicine (TCM) symptom patterns; this approach enables individual treatment by categorizing the signs and symptoms of patients into a series of syndrome concepts^69^: four^48,52,60,64^ were associated with stagnation of the liver or qi, and the remaining one^59^ was a liver-kidney yin deficiency. As control interventions, a total of three types of antidepressants were used: selective serotonin reuptake inhibitors in nine^50,52–54,56,57,60,63,68^, TCA in three^49,55,58^, and flupentixol/melitracen in nine^48,51,59,61,62,64–67^. In most cases, routine care for stroke (RCS) using pharmaceutical anti-platelet, anti-coagulation, and neurotrophic agents, and vasodilators, was performed for both groups. In one study^53^, psychotherapy was performed with the RCS for both groups. The most frequently used outcome was TER in 18 studies^48–52,54–65,67^, followed by HAMD in 15^48,50–53,56,57,59,61–67^, NIHSS in 6^51,53,61,65,67,68^, Barthel index in 4^50,53,60,62^, and China Stroke Scale (CSS) in 3^50,52,57^. Seven different calculation methods of TER were used, and among them, 13^48–52,54–58,60,62,63^ calculated TER based on HAMD, 3^50,57,60^ based on stroke scale, i.e. NIHSS or CSS, 2^65,67^ based on both depression and stroke scale, i.e. HAMD and NIHSS, 2^59,61^ based on clinical symptoms, and the remaining 1 study^64^ was based on both the clinical symptoms and the TCM symptom score. Two studies reported the approval of institutional review board (IRB)^51,68^, and 11 studies reported that they had received consent from the participants^51–53,56,59,60,62,64,66–68^.

**Table 1:** Characteristics of included studies.

Study ID	Sample size (included → analyzed)	Mean age (range) (years)	Diagnostic tool for PSD (severity criteria for inclusion)	Inclusion criteria related to stroke	Pattern identification^※^	(A) Treatment intervention (treatment period)	(B) Control intervention	Outcome and results (post-treatment)	Adverse events	Jadad score
**SGYMT vs. antidepressants**										
Dai^48^	80 (40:40) → 80 (40:40)	(A) 58.5 ± 2.3 (40–73) (B) 57.6 ± 3.1 (42–74)	CCMD-3, C-TCM (NA)	no consciousness disorder, no intellectual disability, no aphasia	liver depression and qi stagnation, heat harassing the heart spirit	(1) SGYMT (2) RCS (28d)	(1) Flupentixol 0.5 mg and Melitracen 10 mg 1 T qd (2) RCS	1. Neurological deficit (without description of the scale): (A) > (B)* 2. TER (HAMD): (A) > (B)* 3. HAMD: (A) > (B)*	(A) none (B) insomnia (2 cases), gastrointestinal reactions (2 cases), increased transaminase (2 cases), blurred vision (1 case) Note. §	3
Huang^49^	65 (38:27) → 65 (38:27)	(A) 56.2 ± 17.9 (NR) (B) 57.4 ± 16.8 (NR)	CCMD (HAMD ≥ 8)	(A) cerebral infarction 24, cerebral hemorrhage 14 (B) cerebral infarction 19, cerebral hemorrhage 8 (CT/MRI)/ no consciousness disorder, no intellectual disability	NA	SGYMT (4w)	Amitriptyline 50–200 mg qd	1. TER (clinical symptom, HAMD): (A) > (B)+	NR	2
Huang^50^	60 (20:20:20) → 60 (20:20:20)	(A) 64.8 ± 7.1 (NR) (B1) 64.4 ± 7.2 (NR) (B2) 65.3 ± 6.9 (NR)	CCMD-3, DSM-IV (NA)	Cerebral infarction or hemorrhage (CT/MRI)	NA	(1) SGYMT (2) RCS (60d)	(B2) (1) Fluoxetine hydrochloride 20 mg 1 T qd (2) RCS	1. TER (HAMD): N.S 2. TER (CSS): (A)>(B2)* 3. HAMD: N.S 4. Barthel index: (A)>(B2)* 5. CSS: (A)<(B2)*	(A) felt that the decoction was difficult to drink, but they still persisted (2 cases) (B2) dry mouth (3 cases), constipation (3 cases), dizziness (1 case) Note. §	2
Huang^51^	78 (38:40) → 78 (38:40)	(A) 58.5 ± 9.4 (55–74) (B) 57.7 ± 9.4 (56–75)	CCMD-3 (unclear)	(A) cerebral infarction 19, cerebral hemorrhage 19 (B) cerebral infarction 22, cerebral hemorrhage 18	NA	(1) SGYMT (2) RCS (8w)	(1) Flupentixol 0.5 mg and Melitracen 10 mg 2 T qd (2) RCS	1. HAMD: (A)<(B) + 2. NIHSS: (A)<(B)* 3. FIM: (A)<(B)* 4. TER (HAMD): (A)>(B)*	(A) none (B) none	3
Liu^52^	60 (30:30) → 60 (30:30)	(A) 65.2 ± 14.2 (45–79) (B) 63.4 ± 10.6 (46–77)	CCMD-3 (24>HAMD≥8)	(A) cerebral infarction 23, cerebral hemorrhage 7 (B) cerebral infarction 21, cerebral hemorrhage 9 (CT/MRI)	liver depression and spleen deficiency	(1) SGYMT (2) RCS (8w)	(1) Fluoxetine hydrochloride 20 mg 1 T qd (2) RCS	1. TER (clinical symptom, HAMD): (A)>(B)* 2. Improvement of clinical symptom: (A)>(B)* (headache), (A)>(B)+ (dizziness, limb numbness, palpitation, insomnia, constipation) 3. HAMD: (A)<(B)* 4. CSS: (A)<(B)+	(A) none (B) excitement, insomnia, dizziness, and gastrointestinal reactions (18 cases)	3
Liu^53^	60 (28:32) → 60 (28:32)	(A) 65.4 ± 8.7 (NR) (B) 63.7 ± 9.3 (NR)	CCMD-2 (HAMD≥8)	Cerebral infarction (CT/MRI)/ no consciousness disorder, no aphasia, no understanding and expression disorder, no serious intellectual disability	NA	(1) SGYMT (2) RCS (3) psychotherapy (28d)	(1) Fluoxetine hydrochloride 20 mg 1 T qd (2) RCS (3) psychotherapy	1. HAMD: N.S 2. NIHSS: N.S 3. Barthel index: N.S 4. Serum levels of IL-1β: (A)<(B)* 5. Serum levels of TNF-α: (A)<(B)*	NR	2
Ta^54^	48 (24:24) → 48 (24:24)	(A) 64.5 ± 6.5 (NR) (B) 65.5 ± 6.1 (NR)	CCMD-3 (NA)	(A) cerebral infarction 16, cerebral hemorrhage 8 (B) cerebral infarction 17, cerebral hemorrhage 7 (CT/MRI)	NA	(1) SGYMT (2) RCS (60d)	(1) Fluoxetine hydrochloride 20 mg 1 T qd (2) RCS	1. TER (clinical symptom, HAMD): N.S	NR	3
Wang^55^	70 (35:35) → 70 (35:35)	(A) NR (42–80) (B) NR (44–79)	CCMD-3 (NA)	(A) cerebral infarction 21, cerebral hemorrhage 14 (B) cerebral infarction 22, cerebral hemorrhage 13 (CT/MRI)	NA	(1) SGYMT (2) RCS (3w)	(1) Amitriptyline 12.5–25 mg tid (2) RCS	1. TER (clinical symptom, HAMD): (A) > (B)*	NR	2
Wang^56^	98 (49:49) → 98 (49:49)	(A) 59.6 ± 5.3 (40–77) (B) 60.1 ± 5.7 (38–76)	CCMD-3 (NA)	(A) cerebral infarction 30, cerebral hemorrhage 19 (B) cerebral infarction 29, cerebral hemorrhage 20/ no consciousness disorder, no language disorder, no severe dementia	NA	(1) SGYMT (2) RCS (4w)	(1) Fluoxetine hydrochloride 20 mg 1 T qd (2) RCS	1. HAMD: N.S 2. MESSS: (A)<(B)* 3. TER (HAMD): N.S	(A) none (B) insomnia (3 cases) Note. §	2
Wu^57^	126 (42:42:42) → 126 (42:42:42)	(A) 59.8 ± 7.8 (NR) (B1) 60.5 ± 8.0 (NR) (B2) 58.6 ± 7.4 (NR)	CCMD-3, C-TCM (NA)	Cerebral infarction (CT/MRI)	NA	(1) SGYMT (2) RCS (3 m)	(B1) (1) Fluoxetine hydrochloride 20 mg 1 T qd (2) RCS	1. HAMD: N.S 2. CSS: (A)<(B1)* 3. TER (HAMD): (A) > (B1)* 4. TER (CSS): (A)>(B1)*	NR	2
Zhang^58^	172 (83:89) → 165 (83:82)	(A) 61.5 ± 7.5 (42–76) (B) 62.3 ± 6.5 (43–79)	CCMD-3 (NA)	(A) cerebral infarction 37, cerebral hemorrhage 46 (B) cerebral infarction 61, cerebral hemorrhage 28 (CT)/ no consciousness disorder, no obvious language disorder	NA	(1) SGYMT (2) RCS (3w)	(1) Amitriptyline 12.5–25 mg tid (2) RCS	1. TER (HAMD): (A)>(B)*	(B) withdrew due to the inability to tolerate the adverse effects of amitriptyline (the number of cases and symptoms were not reported)	3
Zhang^59^	60 (30:30) → 60 (30:30)	(A) 58.7 ± 9.7 (NR) (B) 57.0 ± 5.5 (NR)	CCMD-3 (NA)	Stroke (CT/MRI)/ no consciousness disorder, no aphasia, no intellectual disability	liver-kidney yin deficiency	(1) SGYMT (2) RCS (6w)	(1) Flupentixol 0.5 mg and Melitracen 10 mg 1 T qd (2) RCS	1. HAMD: (A) < (B)+ 2. TER (Clinical symptom): (A)>(B)+	(A) none (B) insomnia (6 cases) Note. §	2
Zhang^60^	134 (68:66) → 134 (68:66)	(A) 65.9 ± 10.4 (NR) (B) 68.1 ± 9.7 (NR)	CCMD-3, IM-TCM, C-TCM (unclear)	Stroke (CT/MRI)/ no consciousness disorder, no language disorder, no communication disorder, no dementia	stagnant qi movement, internal harassment of phlegm-heat	(1) SGYMT (2) RCS (14d)	(1) Escitalopram 20 mg 1 T qd (2) RCS	1. TER (HAMD): (A)>(B)+ 2. TER (NIHSS): N.S 3. Modified Barthel index: (A)>(B)* 4. TCM symptom score: (A) < (B)+	NR	2
**SGYMT + antidepressants vs. antidepressants alone**										
Huang^61^	48 (24:24) → 48 (24:24)	(A) 63.3 ± 4.4 (51–77) (B) 63.9 ± 4.3 (50–78)	C-TCM (NA)	(A) cerebral infarction 11, cerebral hemorrhage 13 (B) cerebral infarction 10, cerebral hemorrhage 14 (CT/MRI)	NA	SGYMT + (B) (28d)	(1) Flupentixol 0.5 mg and Melitracen 10 mg 1 T bid (2) RCS	1. HAMD: (A)<(B)* 2. NIHSS: (A)<(B)* 3. TER (clinical symptoms): (A)>(B)*	NR	2
Lai^62^	68 (34:34) → 68 (34:34)	(A) 58.2 ± 5.8 (52–64) (B) 62.1 ± 6.9 (55–69)	CCMD-3, IM-TCM (HAMD>20)	(A) cerebral infarction 32, cerebral hemorrhage 2 (B) cerebral infarction 33, cerebral hemorrhage 1 (CT/MRI)	NA	SGYMT + (B) (8w)	(1) Flupentixol 0.5 mg and Melitracen 10 mg 2 T qd (2) RCS	1. HAMD: (A)<(B) + 2. Barthel index: (A)>(B)* 3. TER (clinical symptoms, HAMD): (A)>(B)*	NR	2
Li^63^	70 (35:35) → 70 (35:35)	(A) 63.5 ± 6.1 (49–76) (B) 67.5 ± 6.1 (46–81)	CCMD-2-R (NA)	(A) cerebral infarction 27, cerebral hemorrhage 8 (B) cerebral infarction 29, cerebral hemorrhage 6 (CT/MRI)/ no consciousness disorder, no language communication disorder	NA	SGYMT + (B) (8w)	(1) Fluoxetine 20–40 mg qd (2) RCS	1. HAMD: (A) < (B)* 2. Neurological deficit (without description of the scale): (A) < (B)* 3. TER (clinical symptoms, HAMD): (A) > (B)*	NR	3
Li^64^	72 (36:36) → 72 (36:36)	(A) 56.97 ± 10.83 (43–67) (B) 57.06 ± 11.02 (42–68)	CCMD-3 (HAMA≥14, HAMD≥18)	(A) cerebral infarction 24, cerebral hemorrhage 12 (B) cerebral infarction 23, cerebral hemorrhage 13/no consciousness disorder, no aphasia, no cognitive impairment	liver qi depression and phlegm-heat	SGYMT + (B) (4 weeks)	(1) Flupentixol 0.5 mg and Melitracen 10 mg 1 T bid (2) RCS	1. HAMD: (A)<(B)* 2. HAMA: (A)<(B)* 3. TER (clinical symptoms, TCM symptom score): (A) > (B)* 4. TCM symptom score: (A)<(B)*	NR	2
Liu^65^	60 (30:30) → 60 (30:30)	(A) 65.2 ± 7.4 (50–78) (B) 68.5 ± 8.5 (45–81)	CCMD-3 (NA)	(A) cerebral infarction 23, cerebral hemorrhage 7 (B) cerebral infarction 24, cerebral hemorrhage 6 (CT/MRI)/ no consciousness disorder, no language disorder, no intellectual disability	NA	SGYMT + (B) (8w)	(1) Flupentixol 0.5 mg and Melitracen 10 mg 1 T qd-bid (2) RCS	1. HAMD: (A)<(B)* 2. NIHSS: (A)<(B)* 3. TER (HAMD, NIHSS): NR	NR	2
Liu^66^	80 (40:40) → 80 (40:40)	(A) 58.5 ± 2.3 (40–73) (B) 57.6 ± 3.1 (42–69)	Diagnostic guidelines from related societies in China, C-TCM (NA)	(A) cerebral infarction 27, cerebral hemorrhage 13 (B) cerebral infarction 28, cerebral hemorrhage 12 (imaging examination)/ no consciousness disorder, no aphasia, no cognitive impairment	NA	SGYMT + (B) (4w)	(1) Flupentixol 0.5 mg and Melitracen 10 mg 1 T bid (2) RCS	1. TCM symptom score: (A)<(B)+ 2. HAMD: (A) > (B)+ 3. GQOLI-74: (A) > (B)+	NR	3
Wu^67^	82 (41:41) → 82 (41:41)	59.3 ± 3.6 (46–76)	WHO criteria (ICD) (NA)	Cerebral infarction 51, cerebral hemorrhage 31 (CT/MRI)/ no consciousness disorder, no aphasia, no communication disorder	NA	SGYMT + (B) (2 m)	(1) Flupentixol 0.5 mg and Melitracen 10 mg 1 T qd-bid (2) RCS	1. HAMD: (A)<(B)+ 2. NIHSS: (A)<(B)+ 3. TER (clinical symptoms, HAMD, NIHSS): (A) > (B)+	NR	2
Zhang^68^	60 (30:30) → 60 (30:30)	(A) 64.1 ± 5.9 (48–77) (B) 64.5 ± 5.7 (49–78)	CCMD (NA)	(A) cerebral infarction 24, cerebral hemorrhage 6 (B) cerebral infarction 23, cerebral hemorrhage 7 (CT/MRI) / no consciousness disorder	NA	SGYMT + (B) (8w)	(1) Fluoxetine 20–40 mg qd (2) RCS	1. NIHSS: (A) < (B)+	NR	3

^¶^Among three groups in this study, data for the control group undergoing psychotherapy combined with RCS was removed, as this was considered an irrelevant intervention.

^§^Both groups showed no significant abnormality in blood and urine test, kidney function, and electrocardiogram.

^※^An approach of some East Asian traditional medicines, including TCM, which enables individual treatment by categorizing the signs and symptoms of patients into a series of syndrome concepts.

‘*’ and ‘+’ mean significant differences between two groups, p < 0.05 and p < 0.01, respectively. ‘N.S’ means no significant difference between two groups, p > 0.05.

Abbreviations. CCMD, Chinese classification of mental disorders; CSS, China stroke scale; C-TCM, criteria of diagnosis and therapeutic effect of diseases and syndromes in TCM; DSM, diagnostic and statistical manual of mental disorders; FIM, functional independence measure; GQOLI-74, generic quality of life inventory-74; HAMD, Hamilton depression scale; ICD, international classification of diseases; IM-TCM, internal medicine of TCM; MESSS, modified Edinburgh-Scandinavian stroke scale; NA, not applicable; NIHSS, National Institutes of Health stroke scale; NR, not recorded; PSD, post-stroke depression; RCS, routine care for stroke; SGYMT, Sihogayonggolmoryeo-tang; TCM, traditional Chinese medicine; TER, total effective rates; WHO, World Health Organization.

### Methodological quality

**Figure 2:**
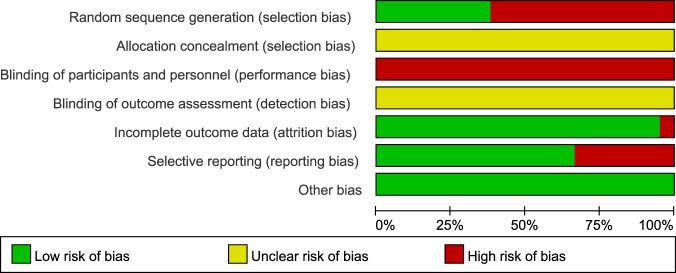
Risk of bias graph for all included studies.

**Figure 3:**
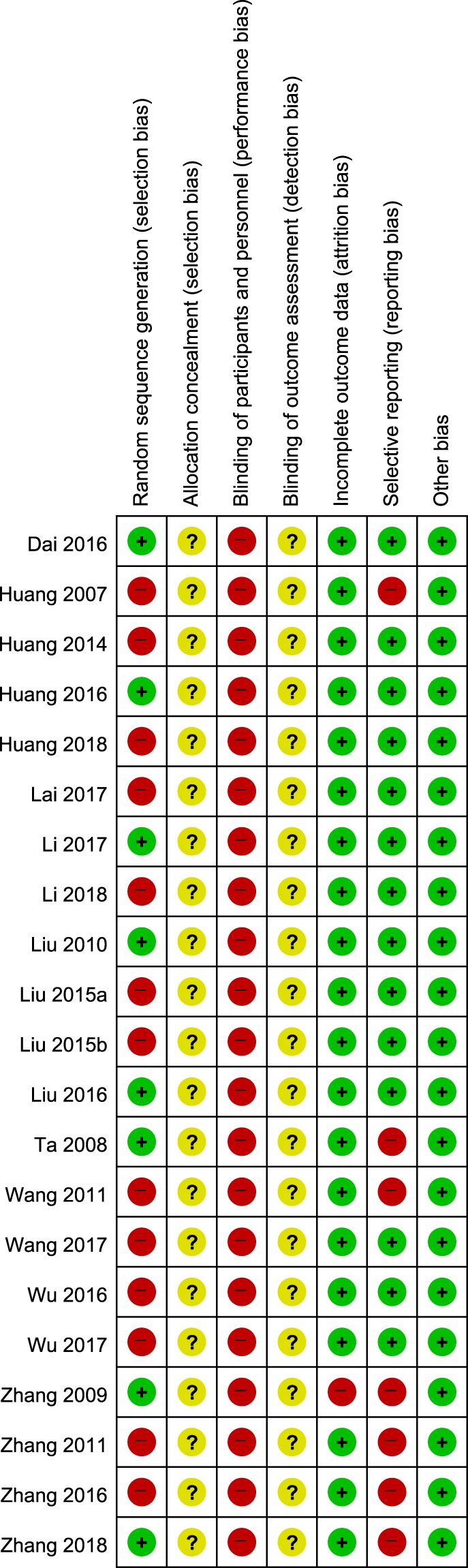
Risk of bias summary for all included studies. Low, unclear, and high risk, respectively, are represented with the following symbols: “+”, “?”, and “−”.

Based on analysis using the Cochrane risk of bias tool, eight studies^48,51,52,54,58,63,66,68^ using appropriate methods of random sequence generation, such as computerized random number tables, were considered to have a low risk of bias on the random sequence generation domain. The remaining 13 studies^49,50,53,55–57,59–62,64,65,67^ were considered to have a high risk of bias because they did not describe their random sequence generation methods. No studies reported allocation concealment, or blinding of participants, personnel, and outcome assessors. The domain of participant and personnel blinding was rated as a high risk of bias in all studies, given that no study used placebos. For 2 studies that reported dropout^54,58^, the domains of incomplete outcome data were rated as low and high risk of bias respectively, according to the processing method for missing data that was intent-to-treat analysis^54^, or per-protocol analysis^58^. None of the included RCTs had published study protocols. Four studies that reported only TER as an outcome^49,54,55,58^, 1 that did not report the result of outcomes that were nonetheless described in the Methods section^59^, 1 that assessed HAMD but did not report the raw data^60^, and 1 that did not report depression-related outcomes^68^, were rated with a high risk of bias in the selective reporting domain. Although we contacted the corresponding authors of 2 of these studies via e-mail to obtain raw data^54,60^, we received no replies. All studies reported no significant baseline difference in demographic data between the two groups, and were rated as having low risk of bias in the other potential sources of bias domains (Figs 2 and 3). Based on the Jadad scale, the mean score was 2.38 (SD 0.50); 8 studies^48,51,52,54,58,63,66,68^ had a total score of 3 and 13^49,50,53,55–57,59–62,64,65,67^ had a total score of 2 (Table 1 and Supplemental Digital Content 2).

### Details of SGYMT administration

The decoction dosage form was used in all studies except for 2 using granules^48,60^. Except for 2 that did not report medication frequency^55,58^, 19 studies instructed patients to take prescriptions twice a day. Twenty-five types of herb were used in addition to 12 types of basic component. Except for *Ginseng Radix* (28.57%) used as a substitute for *Codonopsis Radix*, the remaining 11 basic herbs were used at 61.90–100% frequency in included studies. In particular, *Bupleuri Radix*, *Pinelliae Rhizoma*, and *Fossilia Ossis Mastodi* were used in all studies (all, 100%), and *Poria* and *Ostreae Concha* were used in 20 studies (both, 95.24%). The 25 additional herbs showed 4.76–42.86% frequency of use depending on the type, among which *Curcumae Radix* and *Glycyrrhizae Radix* showed the most frequent with 42.86%, followed by *Astragali Radix*, *Hoelen cum Pini Radix* and *Angelicae Gigantis Radix* at 28.57%, respectively (Supplemental Digital Content 3, which describes the details of SGYMT and herbs added to the original SGYMT formulation).

### SGYMT versus antidepressants

#### Efficacy

**Figure 4:**
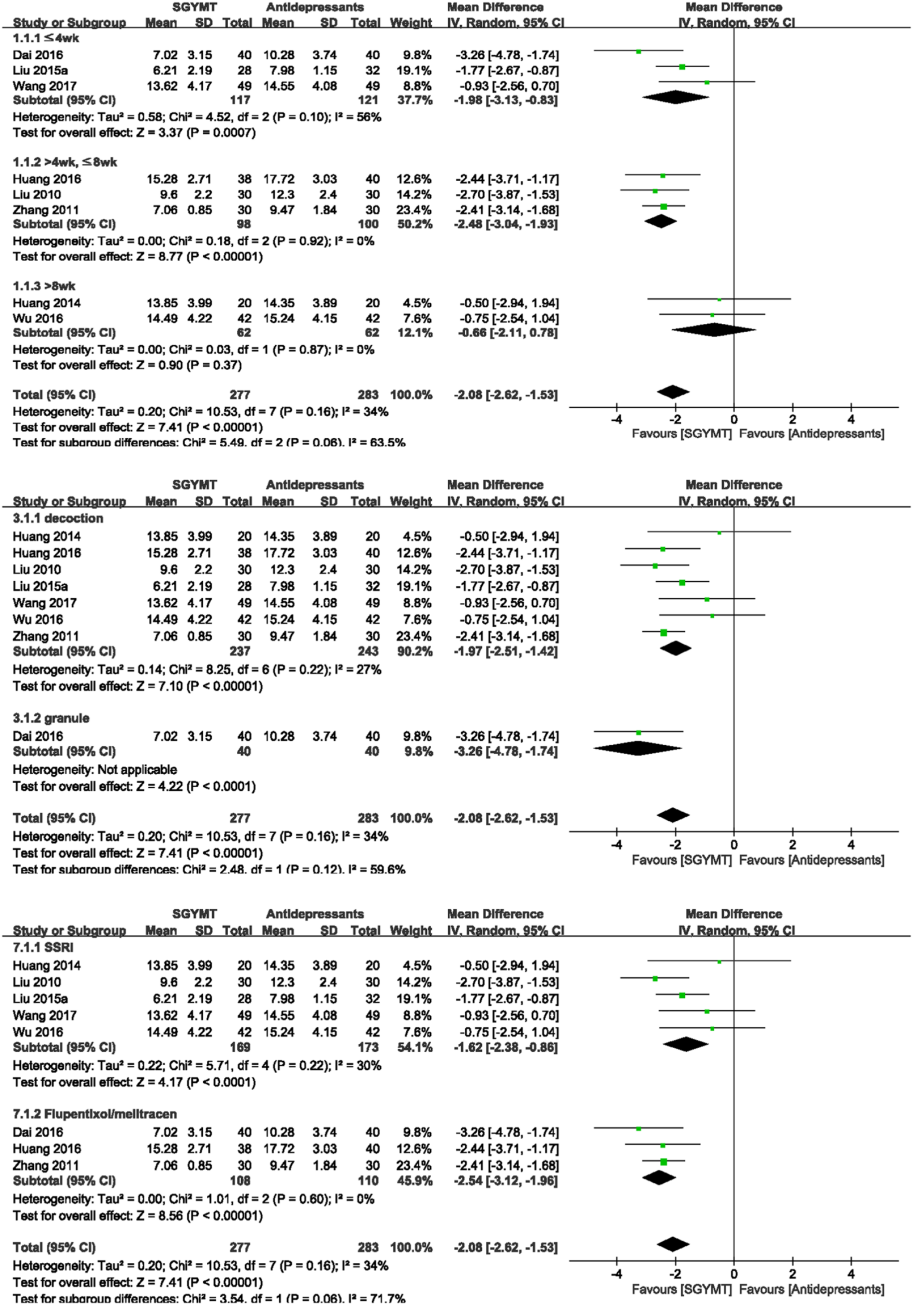
Forest plots for comparison of HAMD scores between SGYMT and pharmaceutical antidepressant groups. Subgroup analysis according to (**a**) treatment period, (**b**) dosage form, and (**c**) types of antidepressants. HAMD, Hamilton Depression Scale; SGYMT, Shihogayonggolmoryeo-tang.

The meta-analysis showed that HAMD scores were significantly lower in the SGYMT group (8 studies^48,50–53,56,57,59^; MD −2.08, 95% CI −2.62 to −1.53, *I* ^2^ = 34%) (Fig. 4), and TERs based on depression scale were higher (11 studies^48–52,54–58,60^; RR 1.11, 95% CI 1.06 to 1.17, *I* ^2^ = 0%) than corresponding scores in the antidepressants group. Subgroup analysis showed that when the treatment period was longer than 8 weeks, these significant between-group differences disappeared for the depression scales including HAMD (2 studies^50,57^; MD −0.66, 95% CI −2.11 to 0.78, *I* ^2^ = 0%), and for TERs based on depression scales (3 studies^50,54,57^; RR 1.05, 95% CI 0.91 to 1.21, *I* ^2^ = 0%). To confirm the robustness of these results, sensitivity analyses were performed after excluding low quality RCTs that had 3 or less low risk of bias on the 7 domains of the risk of bias tool. The superior effectiveness of SGYMT demonstrated by the depression scales including HAMD, and the TER, was consistent within 8 weeks of treatment (Supplemental Digital Content 4).

The neurological functions evaluated by TER based on stroke scale (3 studies^50,54,57^; RR 1.31, 95% CI 1.15 to 1.49, *I* ^2^ = 89%), NIHSS (2 studies^51,53^; MD −0.84, 95% CI −1.40 to −0.29, *I* ^2^ = 19%), and CSS (3 studies^50,52,57^; MD −5.37, 95% CI −6.60 to −4.15, *I* ^2^ = 43%), and the ADL evaluated by the Barthel index (3 studies^50,52,60^; MD 4.30, 95% CI 2.04 to 6.57, *I* ^2^ = 66%) all showed significantly better results in the SGYMT group. In the subgroup analysis, the significant difference between the two groups for TER based on stroke scale disappeared when the treatment period was shorter than 4 weeks (1 study^60^; RR 1.06, 95% CI 0.94 to 1.19) and NIHSS (1 study^53^; MD −0.37, 95% CI −1.37 to 0.63) (Supplemental Digital Content 5, showing forest plots for other outcomes compared between the SGYMT and antidepressant groups).

Wang and Li^56^ and Huang *et al*.^51^ reported modified Edinburgh-Scandinavian stroke scales and functional independence measures respectively as their outcomes, with the SGYMT group showing significantly better results relative to the control group (p < 0.05 for both studies). Moreover, Liu *et al*.^53^ reported significantly lower serum levels of interleukin-1β and tumor necrosis factor-α in the SGYMT group after 28 days of treatment (p < 0.05 for both comparisons).

#### Safety

**Figure 5:**
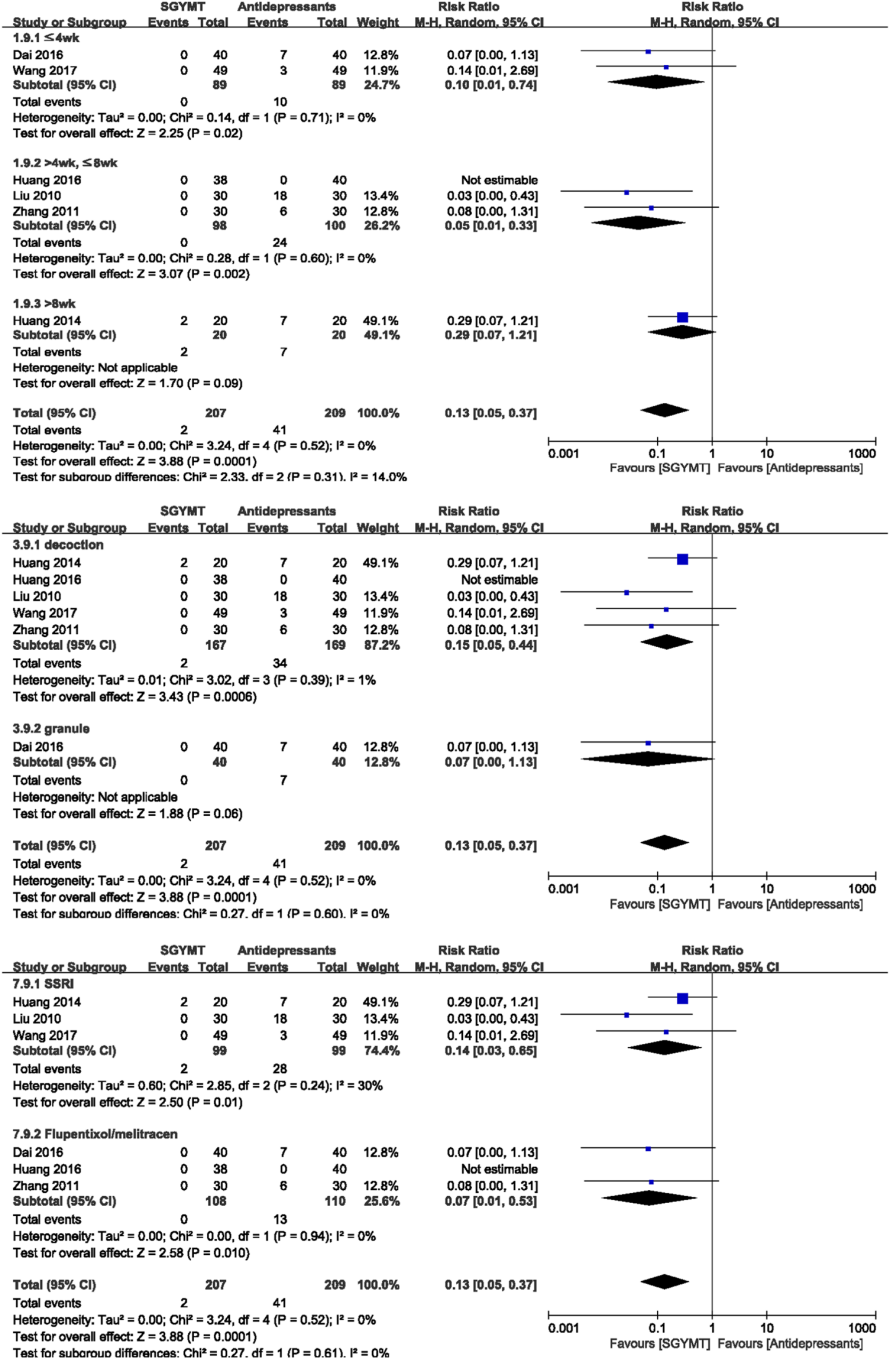
Forest plots for comparison of adverse events between SGYMT and pharmaceutical antidepressant groups. Subgroup analysis according to (**a**) treatment period (**b**) dosage form, and (**c**) types of antidepressants. SGYMT, Shihogayonggolmoryeo-tang.

There were significantly fewer AEs associated with SGYMT (6 studies^48,50–52,56,59^; RR 0.13, 95% CI 0.05 to 0.37, *I* ^2^ = 0%) than with antidepressants (Fig. 5). In the subgroup analysis, significant differences between these two groups disappeared when the treatment period was longer than 8 weeks (1 study^50^; RR 0.29, 95% CI 0.07 to 1.21), or when SGYMT was administered as granules (1 study^48^; RR 0.07, 95% CI 0.00 to 1.13). However, sensitivity analysis performed by excluding low quality RCTs showed no significant difference between two groups when the treatment period was shorter than 4 weeks (1 study^48^; RR 0.07, 95% CI 0.00 to 1.13) or when the type of antidepressant consisted of flupentixol/melitracen (2 studies^48,51^; RR 0.07, 95% CI 0.00 to 1.13) (Supplemental Digital Content 4).

### SGYMT combined with antidepressants versus antidepressants alone

#### Efficacy

**Figure 6:**
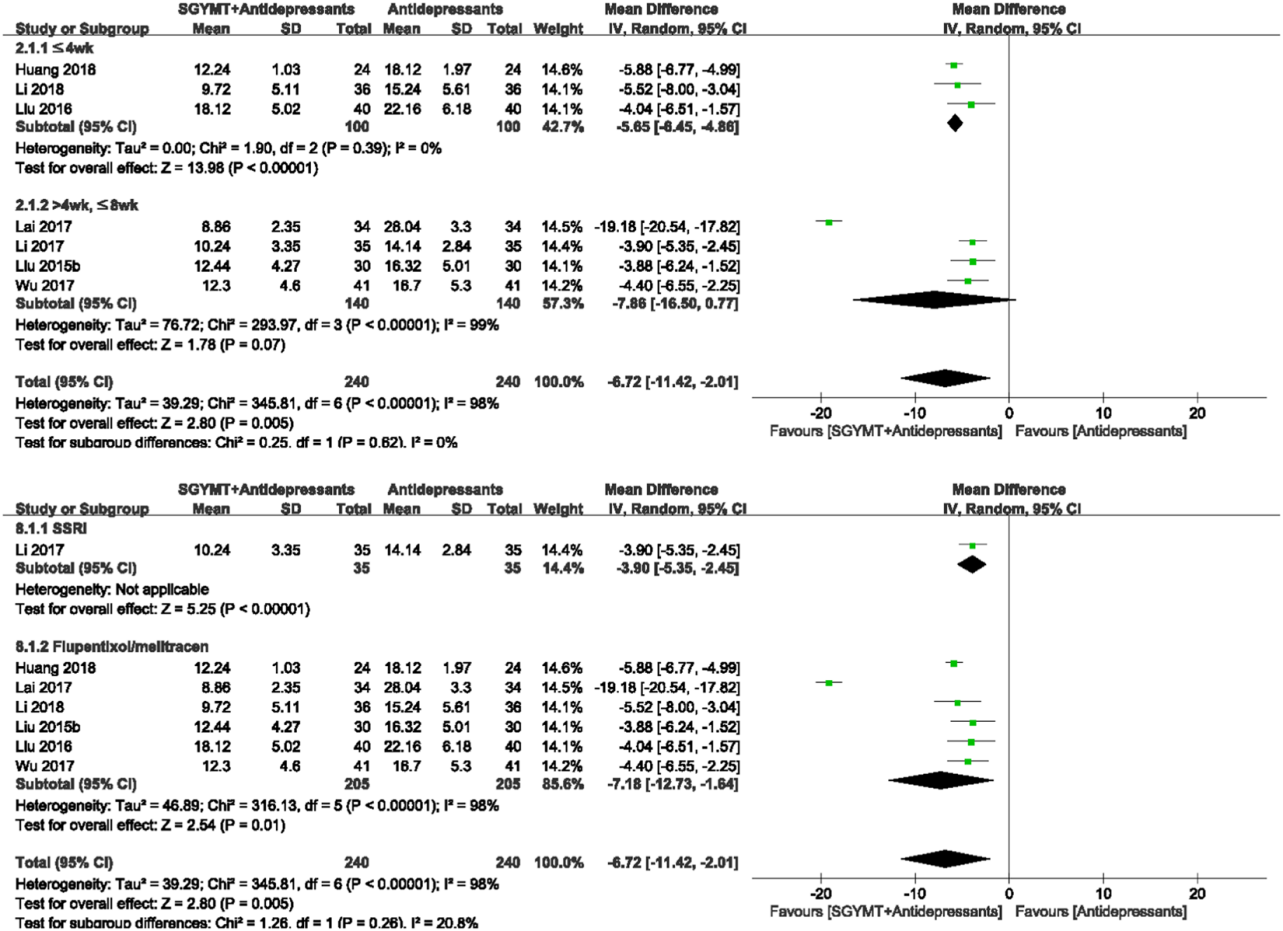
Forest plot for comparison of HAMD score between the SGYMT plus antidepressants group and the antidepressants alone group. Subgroup analysis according to (**a**) treatment period and (**b**) types of antidepressants. HAMD, Hamilton Depression Scale; SGYMT, Shihogayonggolmoryeo-tang.

The meta-analysis showed that the combination therapy group scored significantly lower on the HAMD (7 studies^61–67^; MD = −6.72, 95% CI = −11.42 to −2.01, *I* ^2^ = 98%) (Fig. 6) and NIHSS (4 studies^61,65,67,68^; MD −3.03, 95% CI −3.60 to −2.45, *I* ^2^ = 87%), and showed higher TER based on depression scales (3 studies^62,63,67^; RR 1.66, 95% CI 1.40 to 1.97, *I* ^2^ = 94%) than did the antidepressants alone group (see Supplemental Digital Content 6, showing forest plots comparing other outcomes between SGYMT plus antidepressants and antidepressants only groups).

Interestingly, significant differences in HAMD between treatment groups disappeared when the treatment period was longer than 4 weeks (4 studies^62,63,65,67^; MD −7.86, 95% CI −16.50 to 0.77, *I* ^2^ = 99%) (Fig. 6). Sensitivity analysis performed by excluding low quality RCTs showed that the combination treatment was consistently more effective when the treatment lasted less than 4 weeks (1 study^66^; MD −4.04, 95% CI −6.51 to −1.57). In addition, the extremely high heterogeneity (*I*^2^ = 98%) in the HAMD scores was reduced to 0% as a result of the sensitivity analysis performed by excluding low-quality RCTs (Supplemental Digital Content 4).

Liu and Wang^65^ calculated TER using both depression and stroke scales, and reported that the two groups showed similar efficacies (29/30 for the combination group, 27/30 for the control group, no P-value reported). Lai *et al*.^62^ and Liu^66^ reported the Barthel index and generic quality of life inventory-74 as their outcomes. Using these measures, the combination group showed significantly better results than did the antidepressants alone group (p < 0.05 and p < 0.01, respectively).

#### Safety

No studies reported outcomes related to safety in this comparison.

### Quality of evidence

In the comparison of SGYMT and antidepressants, the qualities of evidence were graded as “Very low” to “Moderate” (Table 2). Meanwhile, in the comparison of SGYMT combined with antidepressants and antidepressants alone, the qualities of evidence were graded as “Very low” to “Moderate” (Table 3). There was no high quality of evidence. The main reason for downgrading was the high risk of bias in the RCTs included in each meta-analysis. In addition, most findings were judged to have low precision because they did not satisfy the optimal sample size and had wide CIs. The indirectness of outcome measure also lowered the quality of evidence.

**Table 2:** Summary of findings: SGYMT compared with antidepressants.

Outcomes		No. participants (RCTs)	Anticipated absolute effects (95% CI)		Quality of evidence (GRADE)	Comments
			Risk with antidepressants	Risk with SGYMT		
**HAMD**	Total	560 (8)	—	MD 2.08 lower (2.62 to 1.53 lower)	⊕⊕◯◯ LOW	Risk of bias (−1) Imprecision (−1)
Subgroup 1	≤4 wk	238 (3)	—	MD 1.98 lower (3.13 to 0.83 lower)	⊕⊕◯◯ LOW	Risk of bias (−1) Imprecision (−1)
	>4 wk, ≤8 wk	198 (3)	—	MD 2.48 lower (3.04 to 1.93 lower)	⊕⊕⊕◯ MODERATE	Risk of bias (−1)
	>8 wk	124 (2)	—	MD 0.66 lower (2.11 lower to 0.78 higher)	⊕⊕◯◯ LOW	Risk of bias (−1) Imprecision (−1)
Subgroup 2	decoction	480 (7)	—	MD 1.97 lower (2.51 to 1.42 lower)	⊕⊕◯◯ LOW	Risk of bias (−1) Imprecision (−1)
	granule	80 (1)	—	MD 3.26 lower (4.78 to 1.74 lower)	⊕⊕◯◯ LOW	Risk of bias (−1) Imprecision (−1)
Subgroup 3	SSRI	342 (5)	—	MD 1.62 lower (2.38 to 0.86 lower)	⊕⊕◯◯ LOW	Risk of bias (−1) Imprecision (−1)
	Flupentixol/melitracen	218 (3)	—	MD 2.54 lower (3.12 to 1.96 lower)	⊕⊕⊕◯ MODERATE	Risk of bias (−1)
**TER (depression scale)**	Total	922 (11)	802 per 1,000	890 per 1,000 (850 to 939)	⊕◯◯◯ VERY LOW	Risk of bias (−1) Indirectness (−1) Imprecision (−1)
Subgroup 1	≤4 wk	612 (6)	823 per 1,000	913 per 1,000 (856 to 971)	⊕⊕◯◯ LOW	Risk of bias (−1) Indirectness (−1)
	>4 wk, ≤8 wk	138 (2)	729 per 1,000	882 per 1,000 (772 to 1,000)	⊕⊕◯◯ LOW	Risk of bias (−1) Indirectness (−1)
	>8 wk	172 (3)	791 per 1,000	830 per 1,000 (720 to 957)	⊕◯◯◯ VERY LOW	Risk of bias (−1) Indirectness (−1) Imprecision (−2)
Subgroup 2	decoction	708 (9)	782 per 1,000	892 per 1,000 (837 to 947)	⊕◯◯◯ VERY LOW	Risk of bias (−1) Indirectness (−1) Imprecision (−1)
	granule	214 (2)	868 per 1,000	929 per 1,000 (833 to 1,000)	⊕⊕◯◯ LOW	Risk of bias (−1) Indirectness (−1)
Subgroup 3	SSRI	464 (6)	801 per 1,000	849 per 1,000 (785 to 905)	⊕◯◯◯ VERY LOW	Risk of bias (−1) Indirectness (−1) Imprecision (−1)
	TCA	300 (3)	799 per 1,000	934 per 1,000 (839 to 1,000)	⊕⊕◯◯ LOW	Risk of bias (−1) Indirectness (−1)
	Flupentixol/melitracen	158 (2)	813 per 1,000	951 per 1,000 (845 to 1,000)	⊕⊕◯◯ LOW	Risk of bias (−1) Indirectness (−1)
**TER (stroke scale)**	Total (SSRI)	258 (3)	680 per 1,000	890 per 1,000 (782 to 1,000)	⊕⊕◯◯ LOW	Risk of bias (−1) Indirectness (−1)
Subgroup 1	≤4 wk	134 (1)	864 per 1,000	915 per 1,000 (812 to 1,000)	⊕⊕◯◯ LOW	Risk of bias (−1) Indirectness (−1)
	>8 wk	124 (2)	484 per 1,000	871 per 1,000 (663 to 1,000)	⊕◯◯◯ VERY LOW	Risk of bias (−1) Indirectness (−1) Imprecision (−1)
Subgroup 2	decoction	124 (2)	484 per 1,000	871 per 1,000 (663 to 1,000)	⊕◯◯◯ VERY LOW	Risk of bias (−1) Indirectness (−1) Imprecision (−1)
	granule	134 (1)	864 per 1,000	915 per 1,000 (812 to 1,000)	⊕⊕◯◯ LOW	Risk of bias (−1) Indirectness (−1)
**NIHSS**	Total (decoction)	138 (2)	—	MD 0.84 lower (1.40 to 0.29 lower)	⊕⊕◯◯ LOW	Risk of bias (−1) Imprecision (−1)
Subgroup 1	≤4 wk	60 (1)	—	MD 0.37 lower (1.37 lower to 0.63 higher)	⊕◯◯◯ VERY LOW	Risk of bias (−1) Imprecision (−2)
	>4 wk, ≤8 wk	78 (1)	—	MD 1.05 lower (1.71 to 0.39 lower)	⊕⊕◯◯ LOW	Risk of bias (−1) Imprecision (−1)
Subgroup 2	SSRI	60 (1)	—	MD 0.37 lower (1.37 lower to 0.63 higher)	⊕◯◯◯ VERY LOW	Risk of bias (−1) Imprecision (−2)
	Flupentixol/melitracen	78 (1)	—	MD 1.05 lower (1.71 to 0.39 lower)	⊕⊕◯◯ LOW	Risk of bias (−1) Imprecision (−1)
**CSS**	Total (decoction/SSRI)	184 (3)	—	MD 5.37 lower (6.60 to 4.15 lower)	⊕⊕⊕◯ MODERATE	Risk of bias (−1)
Subgroup 1	>4 wk, ≤8 wk	60 (1)	—	MD 4.20 lower (5.95 to 2.45 lower)	⊕⊕◯◯ LOW	Risk of bias (−1) Imprecision (−1)
	>8 wk	124 (2)	—	MD 6.50 lower (8.21 to 4.79 lower)	⊕⊕⊕◯ MODERATE	Risk of bias (−1)
**Barthel index**	Total (SSRI)	234 (3)	—	MD 4.30 higher (2.04 to 6.57 higher)	⊕⊕◯◯ LOW	Risk of bias (−1) Imprecision (−1)
Subgroup 1	≤4 wk	194 (2)	—	MD 3.16 higher (0.63 to 5.68 higher)	⊕⊕◯◯ LOW	Risk of bias (−1) Imprecision (−1)
	>8 wk	40 (1)	—	MD 8.99 higher (3.88 to 14.10 higher)	⊕⊕◯◯ LOW	Risk of bias (−1) Imprecision (−1)
Subgroup 2	decoction	100 (2)	—	MD 4.20 higher (0.89 to 7.52 higher)	⊕◯◯◯ VERY LOW	Risk of bias (−1) Imprecision (−2)
	granule	134 (1)	—	MD 4.39 higher (1.29 to 7.49 higher)	⊕⊕⊕◯ MODERATE	Risk of bias (−1)
**AEs**	Total	416 (6)	196 per 1,000	26 per 1,000 (10 to 73)	⊕◯◯◯ VERY LOW	Risk of bias (−1) Imprecision (−2)
Subgroup 1	≤4 wk	178 (2)	112 per 1,000	11 per 1,000 (1 to 83)	⊕◯◯◯ VERY LOW	Risk of bias (−1) Imprecision (−2)
	>4 wk, ≤8 wk	198 (3)	240 per 1,000	12 per 1,000 (2 to 79)	⊕◯◯◯ VERY LOW	Risk of bias (−1) Imprecision (−2)
	>8 wk	40 (1)	350 per 1,000	102 per 1,000 (25 to 424)	⊕⊕◯◯ LOW	Risk of bias (−1) Imprecision (−1)
Subgroup 2	decoction	336 (5)	201 per 1,000	30 per 1,000 (10 to 89)	⊕◯◯◯ VERY LOW	Risk of bias (−1) Imprecision (−2)
	granule	80 (1)	175 per 1,000	12 per 1,000 (0 to 198)	⊕⊕◯◯ LOW	Risk of bias (−1) Imprecision (−1)
Subgroup 3	SSRI	198 (3)	283 per 1,000	40 per 1,000 (8 to 184)	⊕◯◯◯ VERY LOW	Risk of bias (−1) Imprecision (−2)
	Flupentixol/melitracen	218 (3)	118 per 1,000	8 per 1,000 (1 to 63)	⊕◯◯◯ VERY LOW	Risk of bias (−1) Imprecision (−2)

**Abbreviations**. AEs, adverse events; CI, confidence interval; CSS, China stroke scale; GRADE, grading of recommendations assessment, development, and evaluation; HAMD, Hamilton depression scale; MD, mean difference; NA, not applicable; NIHSS, national institutes of health stroke scale; RCT, randomized controlled trial; RR, risk ratio; SGYMT, Sihogayonggolmoryeo-tang; SSRI, selective serotonin reuptake inhibitor; TCA, tricyclic antidepressant; TER, total effective rate.

**Table 3:** Summary of findings: SGYMT combined with antidepressants versus antidepressants alone.

Outcomes		No. participants (RCTs)	Anticipated absolute effects (95% CI)		Quality of evidence (GRADE)	Comments
			Risk with antidepressants alone	Risk with SGYMT plus antidepressants		
**HAMD**	Total (decoction)	480 (7)	—	MD = 6.72 lower (11.42 to 2.01 lower)	⊕⊕⊕◯ MODERATE	Risk of bias (−1)
Subgroup 1	≤4 weeks	200 (3)	—	MD = 5.65 lower (6.45 to 4.86 lower)	⊕⊕⊕◯ MODERATE	Risk of bias (−1)
	>4 weeks, ≤8 weeks	280 (4)	—	MD 7.86 lower (16.50 lower to 0.77 higher)	⊕⊕◯◯ LOW	Risk of bias (−1) Imprecision (−1)
Subgroup 2	SSRI	70 (1)	—	MD = 3.90 lower (5.35 to 2.45 lower)	⊕⊕◯◯ LOW	Risk of bias (−1) Imprecision (−1)
	Flupentixol/melitracen	410 (6)	—	MD = 7.18 lower (12.73 to 1.64 lower)	⊕⊕⊕◯ MODERATE	Risk of bias (−1)
**TER (depression scale)**	Total (>4 wk, ≤8 wk/decoction)	220 (3)	564 per 1,000	936 per 1,000 (789 to 1,000)	⊕⊕◯◯ LOW	Risk of bias (−1) Indirectness (−1)
Subgroup 1	SSRI	70 (1)	771 per 1,000	941 per 1,000 (771 to 1,000)	⊕◯◯◯ VERY LOW	Risk of bias (−1) Indirectness (−1) Imprecision (−1)
	Flupentixol/melitracen	150 (2)	467 per 1,000	933 per 1,000 (728 to 1,000)	⊕⊕◯◯ LOW	Risk of bias (−1) Indirectness (−1)
**NIHSS**	Total (decoction)	250 (4)	—	MD 3.03 lower (3.60 to 2.45 lower)	⊕⊕⊕◯ MODERATE	Risk of bias (−1)
Subgroup 1	≤4 wk	48 (1)	—	MD 2.60 lower (3.35 to 1.85 lower)	⊕⊕◯◯ LOW	Risk of bias (−1) Imprecision (−1)
	>4 wk, ≤8 wk	202 (3)	—	MD 3.62 lower (4.50 to 2.73 lower)	⊕⊕⊕◯ MODERATE	Risk of bias (−1)
Subgroup 2	SSRI	60 (1)	—	MD 6.56 lower (8.13 to 4.99 lower)	⊕⊕◯◯ LOW	Risk of bias (−1) Imprecision (−1)
	Flupentixol/melitracen	190 (3)	—	MD 2.48 lower (3.10 to 1.87 lower)	⊕⊕⊕◯ MODERATE	Risk of bias (−1)

**Abbreviations**. CI, confidence interval; GRADE, grading of recommendations assessment, development, and evaluation; HAMD, Hamilton depression scale; MD, mean difference; NA, not applicable; NIHSS, national institutes of health stroke scale; RCT, randomized controlled trial; RR, risk ratio; SGYMT, Sihogayonggolmoryeo-tang; SSRI, selective serotonin reuptake inhibitor; TER, total effective rate.

### Publication bias

**Figure 7:**
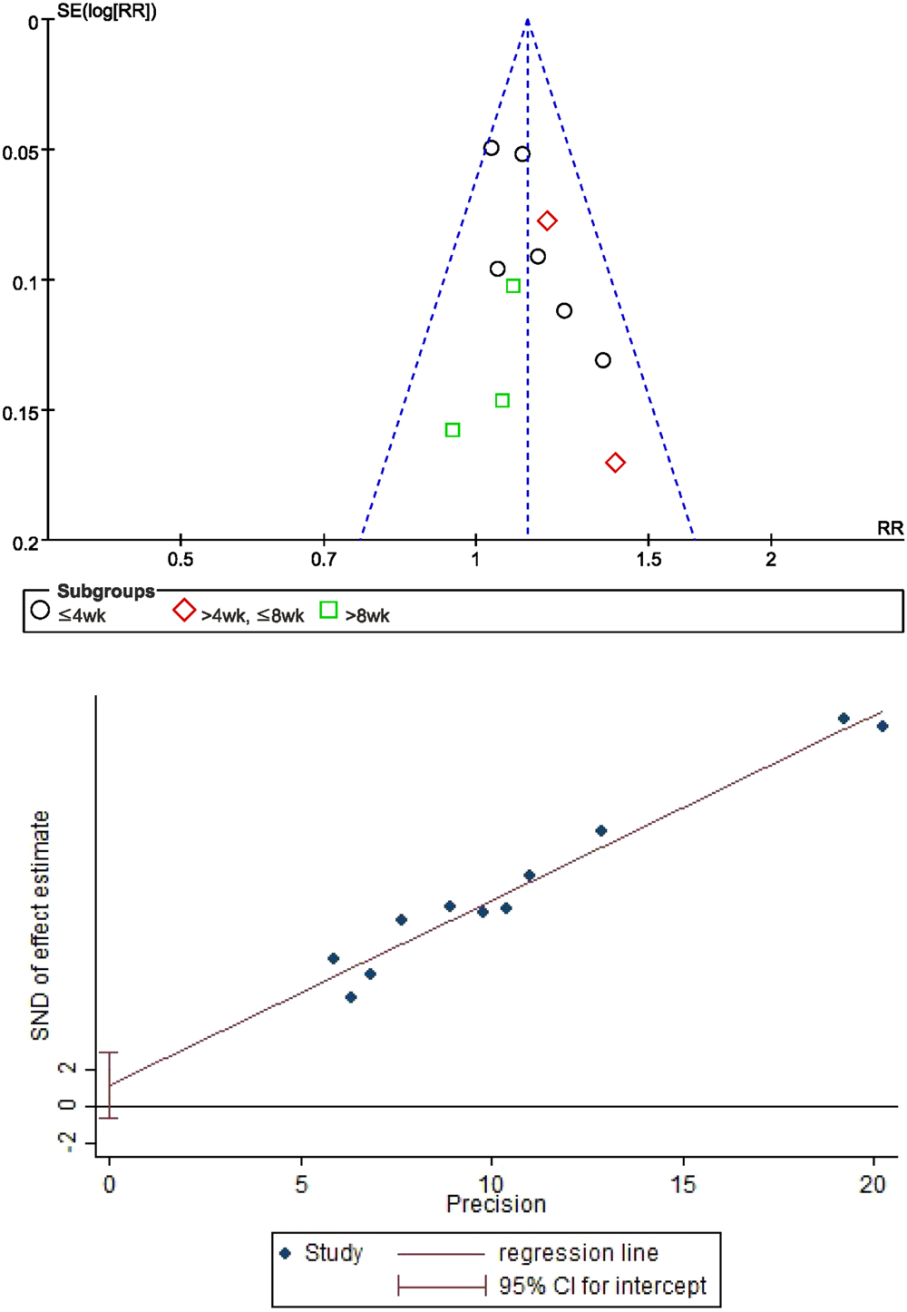
Results of the analysis of publication bias for comparison of TER based on the depression scale between the SGYMT and pharmaceutical antidepressant groups. (**a**) Funnel plot and (**b**) Egger’s regression plot. SGYMT, Shihogayonggolmoryeo-tang; TER, total effective rate.

No evidence of publication bias (distinct asymmetry) emerged from the funnel plots of TER based on depression scales comparing the efficacy of SGYMT with that of antidepressants alone. In addition, publication bias could not be proven using Egger’s method (P value for bias: 0.174) or Begg’s method (continuity corrected Z score: 0.78, continuity corrected P value: 0.436) (Fig. 7).

## Discussion

This review aimed to evaluate the effectiveness and safety of SGYMT as a monotherapy or adjunctive therapy to antidepressants for PSD. A comprehensive search yielded 21 RCTs that were suitable for inclusion in our review.

The findings of our analysis were as follows: (1) In the comparison between SGYMT and antidepressants, relative to pharmaceutical antidepressants, SGYMT monotherapy significantly alleviated depression measured by HAMD (MD −2.08, 95% CI −2.62 to −1.53, *I* ^2^ = 34%), and TER based on depression scale (RR 1.11, 95% CI 1.06 to 1.17, *I* ^2^ = 0%). However, subgroup analysis of treatment periods showed that such differences on HAMD (≤4 weeks: MD −1.98, 95% CI −3.13 to −0.83, *I* ^2^ = 34%; >4 weeks, ≤8 weeks: MD −2.48, 95% CI −3.04 to −1.93, *I* ^2^ = 0%) and TER (≤4 weeks: RR 1.11, 95% CI 1.04 to 1.18, *I* ^2^ = 16%; >4weeks, ≤8weeks: MD 1.21, 95% CI 1.06 to 1.39, *I* ^2^ = 0%) were only evident for treatment periods shorter than 8 weeks, a result consistent with that of the sensitivity analysis performed after exclusion of low quality RCTs. Additionally, the SGYMT group showed significant improvement of neurological functions evaluated by TER based on stroke scale (RR 1.31, 95% CI 1.15 to 1.49, *I* ^2^ = 89%), NIHSS (MD −0.84, 95% CI −1.40 to −0.29, *I*^ 2^ = 19%), and CSS (MD −5.37, 95% CI −6.60 to −4.15, *I* ^2^ = 43%). Differences that emerged from this comparison were sustained when treatment periods were longer than 4 or 8 weeks, for the TER (>8 weeks: RR 1.80, 95% CI 1.37 to 2.37, *I* ^2^ = 0%) and NIHSS outcomes measures (>4weeks, ≤8weeks: MD −1.05, 95% CI −1.71 to −0.39). These results suggest that the effectiveness of SGYMT for treatment of PSD has a different time trajectory relative to that of antidepressants. (2) In the comparison between SGYMT combined with antidepressants and antidepressants alone, the combined treatment also significantly improved depression evaluated by HAMD (MD = −6.72, 95% CI = −11.42 to −2.01, *I* ^2^ = 98%) and TER based on depression scale (RR 1.66, 95% CI 1.40 to 1.97, *I* ^2^ = 94%); however, the benefits assessed using the HAMD were sustained only for treatment periods shorter than 4 weeks (MD = −5.65, 95% CI = −6.45 to −4.86, *I* ^2^ = 0%). These results are consistent with comparisons between SGYMT monotherapy and antidepressants, suggesting that SGYMT may alleviate the symptoms of PSD more rapidly than do pharmaceutical antidepressants. Moreover, the combination treatment group showed more marked improvement of neurological function evaluated by NIHSS (MD −3.03, 95% CI −3.60 to −2.45, *I* ^2^ = 87%) than did the group treated with antidepressants alone. (3) Regarding the safety data, only six RCTs^48,50–52,56,59^, comparing SGYMT with antidepressants reported the incidence of AEs. The SGYMT group showed significantly fewer AEs than did the antidepressants group (RR 0.13, 95% CI 0.05 to 0.37, *I* ^2^ = 0%), regardless of the types of antidepressants compared (SSRI: RR 0.14, 95% CI 0.03 to 0.65, *I*^ *2*^ = 30%; flupentixol/melitracen: RR 0.07, 95% CI 0.01 to 0.53, *I*^ *2*^ = 0%). However, this difference disappeared when treatment periods were longer than 8 weeks (RR 0.29, 95% CI 0.07 to 1.21), or when SGYMT was administered as granules (RR 0.07, 95% CI 0.00 to 1.13). Additionally, sensitivity analysis performed by excluding low quality RCTs showed that the significant difference disappeared when treatment period was shorter than 4 weeks. Altogether, these results suggest that SGYMT may be consistently more effective and safer than antidepressants over treatment periods of 4 to 8 weeks. (4) The methodological quality of the included studies and the strength of evidence were generally poor. The Cochrane risk of bias tool showed that only 8 of 21 included trials used and reported appropriate methods of random sequence generation. Moreover, no studies reported allocation concealment; blinding of participants, personnel, and outcome assessors; or use of placebo designs. Moreover, because none of the studies available for our meta-analysis had previously published a study protocol, their results may be selectively reported and/or biased. We also assessed the quality of RCTs included by using the Jadad scale; the mean score was 2.38, which indicated that the quality of the studies included in this review was generally low. The quality of evidence assessed by the GRADE was “Very low” to “Moderate” and there was no “High” quality evidence. It means that the evidence comparing SGYMT and antidepressants would be significantly improved by future additions of high quality research.

Although a definite conclusion could not be drawn due to the low qualities of included studies and the evidence, our findings suggested the following implications of SGYMT use. First, as an alternative or adjunctive therapy, SGYMT might have antidepressant effects especially within the first 4 to 8 weeks of treatments. Second, SGYMT probably improves neurological function and the ADL for PSD patients, which are difficult to be improved with conventional antidepressants^17^. Third, SGYMT was associated with fewer AEs, especially when administered between 4 and 8 weeks after the start of treatment. However, all these implications are hypothetical and cannot be confirmed by our results.

As a modality of complementary and alternative medicine, HM has been regarded as a potential replacement or supplement for conventional medicine when applied to various pathological conditions including psychiatric disorders such as depression, insomnia, and schizophrenia^70–73^. The underlying mechanism by which SGYMT, one of the famous classical herbal medicines, serves as treatment for PSD is not fully understood; however, for some key herbs of SGYMT, relevant underlying mechanisms have been reported. For example, *Bupleuri Radix*, a key component of the SGYMT prescription, is known to reduce neuro-inflammation^74^ and oxidative stress^75^, and increase concentrations of nerve growth factor and brain-derived neurotrophic factor^76^. All these mechanisms are associated with the etiology of depression. *Scutellariae Radix*, another key component of this prescription, alleviates depression through several complex molecular mechanisms^77^, thereby complementing the action of *Bupleuri Radix*. Some HMs such as Chai Hu Shu Gan San and Xiao Yao San, which include *Bupleuri Radix* as a key component, have significant therapeutic effects on depression^70,78^. Other components of SGYMT, including *Ginseng Radix*, also have antidepressant effects^79–81^. Moreover, the multiple components of HM may exert a complex effect on multiple molecular targets^23^. Thus, HM such as SGYMT may help to improve neurological symptoms in addition to alleviating the symptoms of depression in PSD patients.

The following limitations should be kept in mind when interpreting the results of this meta-analysis. First, because all studies reviewed were conducted in China, general applicability of the results may be limited. Second, the quality of the included studies is generally low, particularly with respect to the lack of placebo-controlled trials. Therefore, the possibility that our study overestimated the effectiveness of SGYMT cannot be ruled out. The low quality of the included studies implies that the reliability of our results is very low. In other words, our results should be interpreted with great caution considering that they may change markedly according to the results of future rigorous research. Furthermore, the popularity of HM in China may have elevated Chinese participants’ expectations of SGYMT. In studies comparing SGYMT combined with antidepressants with antidepressants alone, participants are likely to have high expectations of the former treatment, possibly increasing the placebo effect. Third, in the comparisons within our protocol we planned a subgroup analysis according to the severity of depression, but this could not be carried out because too few studies included criteria assessing the severity of depression. Fourth, only four of the included studies recruited PSD patients with a specific TCM pattern. The TCM pattern can be used in conjunction with the diagnosis of the disease, thereby so-called “disease-syndrome combination” can be used to fully exploit the advantages of the HM^82^, which is advantageous for the individual-specific treatment. Finally, in our review, the control groups of the included studies were prescribed antidepressants regardless of their type, which led to distinct clinical heterogeneity. Although we conducted careful subgroup analyses according to each type of drug, the number of studies included was not sufficient to quantify the comparative effect size of SGYMT compared to each type of antidepressant and to explain the heterogeneity adequately.

Suggestions for future research are as follows. Further high-quality RCTs on the efficacy of SGYMT for reducing PSD are needed, particularly in countries other than China, where wide acceptance of HM for the treatment of PSD may positively bias the results of comparisons with pharmaceutical antidepressants. Accordingly, when planning these studies, it is necessary to consider stratified randomization or post-correction that reflects expectations for HM to avoid potential placebo effects. Moreover, placebo-controlled trials are essential to assess the efficacy and safety of SGYMT objectively. To optimize the use of SGYMT in PSD treatment, future studies should characterize participants in greater detail than was possible in our analysis, particularly the severity of their depression, and their TCM patterns. In particular, individual characteristics are an important component of HM practice, so it is necessary to establish a subgroup of PSD patients with personalized medicine profiles suitable for the administration of SGYMT. TCM patterns may be useful in this selection process. Furthermore, it is important to obtain ethical approval from an IRB before conducting clinical research to protect the dignity, rights, and welfare of research participants, which is in line with World Health Organization guidelines^83^. It is important to explain to the participants the purpose, content, and method of the research, as well as its potential benefits and risks; informed consent should also be obtained from participants in all clinical research studies. In addition, studies using health insurance data in China, Japan, Korea, and Taiwan, where health insurance for HM is applied, may enlarge the database and help specify the indications for SGYMT. Finally, the multi-compound multi-target aspect of HM has potential to contribute to the improvement of both neurological function and depressive symptoms. A comprehensive experimental study of the underlying molecular mechanism of action of SGYMT is needed.

In conclusion, current evidence suggests that SGYMT, either as a monotherapy, or as an adjuvant therapy combined with antidepressants, might have potential benefits for the treatment of PSD. However, since the methodological quality of the included studies was poor and there were no large, placebo-controlled trials to ensure freedom from bias, the results of the meta-analysis may be overestimated; thus, it remains difficult to draw definitive conclusions on this topic. Further well-designed RCTs are needed to confirm these results.

## Supplementary information

Supplemental Digital Content
