# Supplementary material for: Efficacy and safety of Sihogayonggolmoryeo-tang (Saikokaryukotsuboreito, Chai-Hu-Jia-Long-Gu-Mu-Li-Tang) for post-stroke depression: A systematic review and meta-analysis
Source: Sci Rep. 2019 Oct 10;9:14536. doi: 10.1038/s41598-019-51055-6 (PMC6787092; doi:10.1038/s41598-019-51055-6)
Supplement: Supplementary file 1 — Supplemental Digital Content [file 41598_2019_51055_MOESM1_ESM.pdf]

Efficacy and safety of Sihogayonggolmoryeo-tang (Saikokaryukotsuboreito, Chai-Hu-Jia-Long-Gu-Mu-Li-Tang) for post-stroke depression: A systematic review and meta-analysis

Running title: Shihogayonggolmoryeo-tang for post-stroke depression

Chan-Young Kwon[a,b¶], Boram Lee [b¶], Sun-Yong Chung[c], Jong Woo Kim[c], Aesook Shin[a], Ye-yong Choi[a,d], Younghee Yun[a,d], Jungtae Leem[a,e*]

[a] Chung-Yeon Medical Institute, 64 Sangmujungang-ro, Seo-gu, Gwangju 61949, Republic of Korea

[b] Department of Clinical Korean Medicine, Graduate School, Kyung Hee University, 26 Kyung Hee Dae-ro, Dongdaemun-gu, Seoul 02447, Republic of Korea

[c] Department of Korean Medicine, Kyung Hee University Korean Medicine Hospital at Gangdong, 892 Dongnam-ro, Gangdong-gu, Seoul 05278, Republic of Korea

[d] Research and Development Institute, CY Pharma Co., 404 Nonhyeon-ro, Gangnam-gu, Seoul 06224, Republic of Korea

[e] Dongshin Korean Medicine Hospital, 351 Omok-ro, Yangcheon-gu, Seoul 07999, Republic of Korea

E-mail address

Chan-Young Kwon: beanalogue@naver.com

Boram Lee: qhfka9357@naver.com

Sun-Yong Chung: lovepwer@khu.ac.kr

Jong Woo Kim: aromaqi@khu.ac.kr

Aesook Shin: aesook.shin@gmail.com

Ye-yong Choi: choiyy@cypharm.kr

Younghee Yun: allergycosmetic@gmail.com

Jungtae Leem: julcho@naver.com

*** Corresponding author**

Jungtae Leem, MD (DKM), PhD

Dongshin Korean Medicine Hospital, 351 Omok-ro, Yangcheon-gu, Seoul 07999, Republic of Korea

Telephone: +82-2-9640-2902; Fax: +82-2-2640-2727; E-mail: julcho@naver.com

¶ These authors contributed equally to this work (co-first authors).

Figure Legends

Figure 1. PRISMA flow chart of the study selection process.

Moher D, et al. Preferred reporting items for systematic reviews and meta-analyses: the PRISMA statement. PLoS Med 2009;6(7):e1000097

Figure 2. Risk of bias graph for all included studies.

Figure 3. Risk of bias summary for all included studies.
Low, unclear, and high risk, respectively, are represented with the following symbols: "+", "?", and "-"

Figure 4. Forest plots for comparison of HAMD scores between SGYMT and pharmaceutical antidepressant groups.

Subgroup analysis according to (a) treatment period, (b) dosage form, and (c) types of antidepressants.
HAMD, Hamilton Depression Scale; SGYMT, Shihogayonggolmoryeo-tang.

Figure 5. Forest plots for comparison of adverse events between SGYMT and pharmaceutical antidepressant groups.

Subgroup analysis according to (a) treatment period (b) dosage form, and (c) types of antidepressants.
SGYMT, Shihogayonggolmoryeo-tang.

Figure 6. Forest plot for comparison of HAMD score between the SGYMT plus antidepressants group and the antidepressants alone group.

Subgroup analysis according to (a) treatment period and (b) types of antidepressants.
HAMD, Hamilton Depression Scale; SGYMT, Shihogayonggolmoryeo-tang.

Figure 7. Results of the analysis of publication bias for comparison of TER based on the depression scale between the SGYMT and pharmaceutical antidepressant groups.

(a) Funnel plot and (b) Egger's regression plot.
SGYMT, Shihogayonggolmoryeo-tang; TER, total effective rate.

Supplemental Digital Content

Supplemental Digital Content 1. Search strategies used in each database.

Supplemental Digital Content 2. Jadad scores of the included studies.

Supplemental Digital Content 3. Details of SGYMT and herbs added to the original SGYMT formulation.
SGYMT, Shihogayonggolmoryeo-tang.

Supplemental Digital Content 4. Results of sensitivity analysis after removal of studies with high risk of bias.

Supplemental Digital Content 5. Forest plots for other outcomes in comparison between SGYMT and antidepressants.
SGYMT, Shihogayonggolmoryeo-tang.

Supplemental Digital Content 6. Forest plots for other outcomes in comparison between SGYMT plus antidepressants and antidepressants alone.
SGYMT, Shihogayonggolmoryeo-tang.

Supplemental Digital Content 7. Results of meta-analysis using pre-registered fixed-effects models.

Supplemental Digital Content 8. PRISMA 2009 checklist.

Supplemental digital content 1. Search strategies used in each database

MEDLINE via PubMed

	Searches	Results
#1	"depressive disorder"[MeSH Terms] OR "depression"[MeSH Terms] OR depressive OR depression	432461
#2	"stroke"[MeSH Terms] OR stroke	317983
#3	Chai-Hu-Jia-Long-Gu-Mu-Li-Tang OR Chai-Hu-Jia-Long-Gu-Mu-Li-Wan OR Chai-Hu-Jia-Long-Gu-Mu-Li-Pian OR Saikokaryukotsuboreitou OR Saikokaryukotsuboreito OR Sihogayonggolmoryeo-tang	48
#4	#1 AND #2 AND #3	**1**

EMBASE via Elsevier

	Searches	Results
#1	“depression”/exp OR “depression”	683570
#2	Depressive	149196
#3	“cerebrovascular disease”/exp OR “cerebrovascular disease”	688478
#4	“stroke patient”/exp OR “stroke patient”	29406
#5	Stroke	428235
#6	“chai hu jia long gu mu li tang” OR “chai hu jia long gu mu li wan” OR “chai hu jia long gu mu li pian” OR saikokaryukotsuboreitou OR saikokaryukotsuboreito OR “sihogayonggolmoryeo tang”	30
#7	(#1 OR #2) AND (#3 OR #4 OR #5) AND #6	**2**

CENTRAL

	Searches	Results
#1	MeSH descriptor: [depressive disorder] explode all trees	10751
#2	MeSH descriptor: [depression] explode all trees	10317
#3	depressive OR depression	71508
#4	MeSH descriptor: [stroke] explode all trees	8301
#5	stroke	49784
#6	Chai-Hu-Jia-Long-Gu-Mu-Li-Tang OR Chai-Hu-Jia-Long-Gu-Mu-Li-Wan OR Chai-Hu-Jia-Long-Gu-Mu-Li-Pian OR Saikokaryukotsuboreitou OR Saikokaryukotsuboreito OR	6

	Sihogayonggolmoryeo-tang	
#7	(#1 OR #2 OR #3) AND (#4 OR #5) AND #6 in Trials	**0**

AMED via EBSCO

	Searches	Results
#1	depressive disorder[SU]	2378
#2	depression[SU]	1444
#3	depressive[TX] OR depression[TX]	7435
#4	stroke[SU]	3228
#5	stroke[TX]	8776
#6	Chai-Hu-Jia-Long-Gu-Mu-Li-Tang[TX] OR Chai-Hu-Jia-Long-Gu-Mu-Li-Wan[TX] OR Chai-Hu-Jia-Long-Gu-Mu-Li-Pian[TX] OR Saikokaryukotsuboreitou[TX] OR Saikokaryukotsuboreito[TX] OR Sihogayonggolmoryeo-tang[TX]	0
#7	(#1 OR #2 OR #3) AND (#4 OR #5) AND #6	**0**

CINAHL via EBSCO

	Searches	Results
#1	depressive disorder[MH]	0
#2	depression[MH]	93455
#3	depressive[TX] OR depression[TX]	245348
#4	stroke[MH]	60640
#5	stroke[TX]	169191
#6	Chai-Hu-Jia-Long-Gu-Mu-Li-Tang[TX] OR Chai-Hu-Jia-Long-Gu-Mu-Li-Wan[TX] OR Chai-Hu-Jia-Long-Gu-Mu-Li-Pian[TX] OR Saikokaryukotsuboreitou[TX] OR Saikokaryukotsuboreito[TX] OR Sihogayonggolmoryeo-tang[TX]	12
#7	(#1 OR #2 OR #3) AND (#4 OR #5) AND #6	**0**

PsycARTICLES via ProQuest

	Searches	Results
#1	mesh(depressive disorder)	2104

#2	mesh(depression)	2581
#3	depressive OR depression	46796
#4	mesh(stroke)	66
#5	stroke	5720
#6	Chai-Hu-Jia-Long-Gu-Mu-Li-Tang OR Chai-Hu-Jia-Long-Gu-Mu-Li-Wan OR Chai-Hu-Jia-Long-Gu-Mu-Li-Pian OR Saikokaryukotsuboreitou OR Saikokaryukotsuboreito OR Sihogayonggolmoryeo-tang	0
#7	(#1 OR #2 OR #3) AND (#4 OR #5) AND #6	**0**

OASIS

	Searches	Results
#1	(뇌졸중 OR 중풍) AND 우울 AND 시호가용골모려탕	**0**

KISS

	Searches	Results
#1	뇌졸중 AND 우울 AND 시호가용골모려탕	**0**
#2	중풍 AND 우울 AND 시호가용골모려탕	**0**

RISS

	Searches	Results
#1	뇌졸중 AND 우울 AND 시호가용골모려탕	**0**
#2	중풍 AND 우울 AND 시호가용골모려탕	**0**

KMbase

	Searches	Results
#1	뇌졸중 AND 우울 AND 시호가용골모려탕	**0**
#2	중풍 AND 우울 AND 시호가용골모려탕	**0**

KCI

	Searches	Results
#1	뇌졸중 AND 우울 AND 시호가용골모려탕	**0**
#2	중풍 AND 우울 AND 시호가용골모려탕	**0**

CNKI

	Searches	Results
#1	(SU='中风'+'脑卒中') AND (SU='抑郁'+'忧郁') AND (SU='柴胡加龙骨牡蛎汤'+'柴胡龙骨牡蛎汤')	**43**

Wanfang data

	Searches	Results
#1	(“中风” + “脑卒中”) * (“抑郁” + “忧郁”) * (“柴胡加龙骨牡蛎汤” + “柴胡龙骨牡蛎汤”)	**55**

Supplementary digital content 2. Jadad score of the included studies

Study	Total scores	Q1	Q2	Q4	Q5	Q6
Dai 2016 (48)	3	1	1	0	0	1
Huang 2007 (49)	2	1	0	0	0	1
Huang 2014 (50)	2	1	0	0	0	1
Huang 2016 (51)	3	1	1	0	0	1
Liu 2010 (52)	3	1	1	0	0	1
Liu 2015a (53)	2	1	0	0	0	1
Ta 2008 (54)	3	1	1	0	0	1
Wang 2011 (55)	2	1	0	0	0	1
Wang 2017 (56)	2	1	0	0	0	1
Wu 2016 (57)	2	1	0	0	0	1
Zhang 2009 (58)	3	1	1	0	0	1
Zhang 2011 (59)	2	1	0	0	0	1
Zhang 2016 (60)	2	1	0	0	0	1
Huang 2018 (61)	2	1	0	0	0	1
Lai 2017 (62)	2	1	0	0	0	1
Li 2017 (63)	3	1	1	0	0	1
Li 2018 (64)	2	1	0	0	0	1
Liu 2015b (65)	2	1	0	0	0	1
Liu 2016 (66)	3	1	1	0	0	1
Wu 2017 (67)	2	1	0	0	0	1
Zhang 2018 (68)	3	1	1	0	0	1

Q1: Was the study described as randomized? (add 1 point)

Q2: Was the method of randomisation in the paper appropriate? (add 1 point)

or inappropriate? (deduct 1 point)

Q3: Was the study described as double blind? (add 1 point)

Q4: Was the method of blinding in the paper appropriate? (add 1 point)

or inappropriate? (deduct 1 point)

Q5: Was there a description of withdrawals and dropouts? (add 1 point)

Supplemental digital content 3. The details of SGYMT and additional herbs on basic SGYMT

(1) Summarized details of SGYMT use for all studies

**Study**	**Dosage form**	**Administration duration and frequency**	**Dosages of basic components per day (g)**											
			***Bupleuri Radix* (柴胡)**	***Pinelliae Rhizoma* (半夏)**	***Ramulus Cinnamomi* (桂枝)**	***Poria* (茯苓)**	***Scutellariae Radix* (黃芩)**	***Jujubae Fructus* (大棗)**	***Ginseng Radix* (人蔘)**	***Codonopsis Radix* (黨參)**	***Ostreae Concha* (牡蠣)**	***Fossilia Ossis Mastodi* (龍骨)**	***Zingiberis Rhizoma Recens* (生薑)**	***Rhei Rhizoma* (大黃)**
**SGYMT vs. antidepressants**														
Dai 2016 (48)	granule	28d, bid	12	9		10	10			10	30	30	6	6
Huang 2007 (49)	decoction	4w, bid	12	10	10	10	10			15	30	30		6
Huang 2014 (50)	decoction	60d, bid	12	12	9	12	12	Six dates	9		30	30	Three pieces	6
Huang 2016 (51)	decoction	8w, bid	20	15		15		Six dates		20	30	30		
Liu 2010 (52)	decoction	8w, bid	12	10	10	10	10	Ten dates		15	30	30	10	6
Liu 2015a (53)	decoction	28d, bid	15	9	10	20	10	Twenty dates		15	15	15	10	5
Ta 2008 (54)	decoction	60d, bid	15	15	10		10	Four dates	6		30	30	Three pieces	5
Wang 2011 (55)	decoction	3w, NR	12	12	10	10	15		10		20	20	10	10
Wang 2017 (56)	decoction	4w, bid	12	12	9	12	9	Six dates		12	30	30	Three pieces	6
Wu 2016¶ (57)	decoction	3m, bid	12	12	9	12	12	Six dates	9		30	30	Three pieces	6
Zhang 2009 (58)	decoction	3w, NR	12	12	10	10	15		10		20	20	10	10
Zhang 2011 (59)	decoction	6w, bid	12	10	10	10	10	Six dates	10		30	30	10	6
Zhang 2016 (60)	granule	14d, bid	12	12	10	30	10	10		10	30	30	10	6
**SGYMT + antidepressants vs. antidepressants alone**														

Huang 2018 (61)	decoction	28d, bid	12	9	12	30	15				20	20		6
Lai 2017 (62)	decoction	8w, bid	10	10	10	20	10	Twelve dates		10	30	30	10	6
Li 2017 (63)	decoction	8w, bid	20	15	10	15		Six dates		20		30	30	
Li 2018 (64)	decoction	4w, bid	15	10	10	13	10				18	18		5
Liu 2015b (65)	decoction	8w, bid	20	15	10	15		Six dates		20	30	30		
Liu 2016 (66)	decoction	4w, bid	15	10	15	10		10		20	30	30		
Wu 2017 (67)	decoction	2m, bid	20	15		15		Six dates		20	30	30		
Zhang 2018 (68)	decoction	8w, bid	20	15	10	15		Six dates		20	30	30		
Frequency (%)			100	100	85.71	95.24	71.43	71.43	28.57	61.90	95.24	100	61.90	71.43
Mean dosage (g)¶			14.38	11.86	10.22	14.7	11.2	13.3333	9	15.9231	27.15	27.29	11.7778	6.33

¶ The mean dosages were calculated excluding dates and pieces.

Abbreviations. bid, bi-daily; NR, not reported; SGYMT, Sihogayonggolmoryeo-tang.

(2) Use of additional herbs on basic SGYMT

Study	**Dosages of additional components for 1 day (g)**																							
	Magnetitum (磁石)	*Paeoniae Radix Rubra* (赤芍藥)	*Moutan Cortex* (牡丹皮)	*Zizyphi Spinosae Semen* (酸棗仁)	*Polygalae Radix* (遠志)	*Acori Graminei Rhizoma* (石菖蒲)	*Curcumae Radix* (鬱金)	*Paeoniae Radix Alba* (白芍藥)	*Aurantii Immaturus Fructus* (枳實)	*Cyperi Rhizoma* (香附子)	*Glycyrrhizae Radix* (甘草)	*Astragali Radix* (黃芪)	*Hoelen cum Pini Radix* (茯神)	*Pheretimae Corpus* (地龍)	*Chinemydis Carapax* (龜板)	*Salviae Miltiorrhizae Radix* (丹蔘)	*Glehniae Radix* (防風)	*Liriopes Radix* (麥門冬)	*Angelicae Gigantis Radix* (當歸)	*Rehmanniae Radix* (生地黃)	*Lycii Fructus* (枸杞子)	*Toosendan Fructus* (川楝子)	*Margaritifera Usta Concha* (珍珠母)	*Fossilia Mastodi Dentis* (龍齒)	*Gardeniae Fructus* (梔子)
**SGYMT vs. antidepressants**																								
Dai 2016 (48)	30	10	10	30																				
Huang 2007 (49)					10	12	12	20	10	10	6													
Huang 2014 (50)																								
Huang 2016 (51)							15				5	30	15	10										

Liu 2010 (52)	30				10	10									10										
Liu 2015a (53)																									
Ta 2008 (54)											9		10												
Wang 2011 (55)						20	20																		
Wang 2017 (56)																									
Wu 2016 (57)																									
Zhang 2009 (58)						20	20									20									
Zhang 2011 (59)																	10	10	10	20	10	6			
Zhang 2016 (60)																									
**SGYMT + antidepressants vs. antidepressants**																									
Huang 2018 (61)											6														
Lai 2017 (62)	30																						30	20	
Li 2017 (63)							15				10	30	15	10					15						
Li 2018 (64)										12	6														8
Liu 2015b (65)							15				10	30	15	10					15						
Liu 2016 (66)							15				10	20		10					20						
Wu 2017 (67)							15				10	30	15						15						

Zhang 2018 (68)							15					30	15	10					15						
Frequency (%)	14.29	4.76	4.76	4.76	9.52	19.05	42.86	4.76	4.76	9.52	42.86	28.57	28.57	23.81	4.76	4.76	4.76	4.76	28.57	4.76	4.76	4.76	4.76	4.76	4.76
Mean dosage (g)	30	10	10	30	10	15.5	15.7778	20	10	11	8	28.3333	14.1667	10	10	20	10	10	15	20	10	6	30	20	8

Abbreviations. SGYMT, Sihogayonggolmoryeo-tang.

Supplemental digital content 4. Results of sensitivity analysis after removal of studies with high risk of bias

Outcomes		RCT	Sample size	RR or MD	95% CI	I^2 value	Z value	P value
**SGYMT vs. antidepressants**								
**HAMD**	Total	3	218	MD -2.75	-3.49, -2.00	0	7.20	<0.00001
Subgroup 1	≤4 wk	1	80	MD -3.26	-4.78, -1.74	NA	4.22	<0.0001
	>4 wk, ≤8 wk	2	138	MD -2.58	-3.44, -1.72	0	5.88	<0.00001
Subgroup 2	decoction	2	138	MD -2.58	-3.44, -1.72	0	5.88	<0.00001
	granule	1	80	MD -3.26	-4.78, -1.74	NA	4.22	<0.0001
Subgroup 3	SSRI	1	60	MD -2.70	-3.87, -1.53	NA	4.54	<0.00001
	Flupentixol/melitracen	2	158	MD -2.78	-3.76, -1.80	0	5.59	<0.00001
**TER (depression scale)**	Total	3	218	RR 1.22	1.08, 1.37	0	3.24	0.001
Subgroup 1	≤4 wk	1	80	RR 1.16	0.97, 1.38	NA	1.60	0.11
	>4 wk, ≤8 wk	2	138	RR 1.26	1.07, 1.47	0	2.83	0.005
Subgroup 2	decoction	2	138	RR 1.26	1.07, 1.47	0	2.83	0.005
	granule	1	80	RR 1.16	0.97, 1.38	NA	1.60	0.11
Subgroup 3	SSRI	1	60	RR 1.39	1.00, 1.94	NA	1.93	0.05
	Flupentixol/melitracen	2	158	RR 1.17	1.04, 1.31	0	2.60	0.009
**AEs**	Total	3	218	RR 0.04	0.01, 0.28	0	3.24	0.001
Subgroup 1	≤4 wk	1	80	RR 0.07	0.00, 1.13	NA	1.88	0.06
	>4 wk, ≤8 wk	2	138	RR 0.03	0.00, 0.43	NA*	2.56	0.01
Subgroup 2	decoction	2	138	RR 0.03	0.00, 0.43	NA*	2.56	0.01
	granule	1	80	RR 0.07	0.00, 1.13	NA	1.88	0.06
Subgroup 3	SSRI	1	60	RR 0.03	0.00, 0.43	NA	2.56	0.01
	Flupentixol/melitracen	2	158	RR 0.07	0.00, 1.13	NA*	1.88	0.06
**SGYMT + antidepressants vs. antidepressants alone**								
**HAMD**	Total	2	150	MD -3.94	-5.19, -2.68	0	6.16	<0.00001
Subgroup 1	≤4 wk	1	80	MD -4.04	-6.51, -1.57	NA	3.21	0.001
	>4 wk, ≤8 wk	1	70	MD -3.90	-5.35, -2.45	NA	5.25	<0.00001
Subgroup 2	SSRI	1	70	MD -3.90	-5.35, -2.45	NA	5.25	<0.00001
	Flupentixol/melitracen	1	80	MD -4.04	-6.51, -1.57	NA	3.21	0.001
**TER (depression scale)**	Total (>4 wk, ≤8 wk/SSRI)	1	70	RR 1.22	1.00, 1.49	NA	1.99	0.05

*In one of the two studies, it was impossible to calculate the risk ratio because both experimental and control groups reported no adverse events. Therefore, heterogeneity evaluation through I^2 was impossible.

Abbreviations. AEs, adverse events; CI, confidence interval; HAMD, Hamilton depression scale; MD, mean difference; NA, not applicable; RCT, randomized controlled trial; RR, risk ratio; SGYMT, Sihogayonggolmoryeo-tang; SSRI, selective serotonin reuptake inhibitor; TER, total effective rate

Supplemental digital content 5. Forest plots for comparison of SGYMT versus Antidepressants. Other outcomes.

(a) TER based on depression scale. Subgroup analysis according to treatment period

Study or Subgroup	SGYMT Events	SGYMT Total	Antidepressants Events	Antidepressants Total	Weight	Risk Ratio M-H, Random, 95% CI
**1.2.1 ≤4wk**						
Dai 2016	37	40	32	40	7.9%	1.16 [0.97, 1.38]
Huang 2007	36	38	19	27	3.8%	1.35 [1.04, 1.74]
Wang 2011	32	35	26	35	5.2%	1.23 [0.99, 1.53]
Wang 2017	41	49	39	49	7.1%	1.05 [0.87, 1.27]
Zhang 2009	79	83	70	82	24.2%	1.11 [1.01, 1.23]
Zhang 2016	64	68	60	66	26.8%	1.04 [0.94, 1.14]
**Subtotal (95% CI)**		**313**		**299**	**75.0%**	**1.11 [1.04, 1.18]**
Total events	289		246			
Heterogeneity: Tau² = 0.00; Chi² = 5.93, df = 5 (P = 0.31); I² = 16%						
Test for overall effect: Z = 3.05 (P = 0.002)						
**1.2.2 >4wk, ≤8wk**						
Huang 2016	37	38	33	40	10.9%	1.18 [1.01, 1.37]
Liu 2010	25	30	18	30	2.3%	1.39 [1.00, 1.94]
**Subtotal (95% CI)**		**68**		**70**	**13.1%**	**1.21 [1.06, 1.39]**
Total events	62		51			
Heterogeneity: Tau² = 0.00; Chi² = 0.98, df = 1 (P = 0.32); I² = 0%						
Test for overall effect: Z = 2.75 (P = 0.006)						
**1.2.3 >8wk**						
Huang 2014	17	20	16	20	3.1%	1.06 [0.80, 1.41]
Ta 2008	18	24	19	24	2.6%	0.95 [0.70, 1.29]
Wu 2016	36	42	33	42	6.2%	1.09 [0.89, 1.33]
**Subtotal (95% CI)**		**86**		**86**	**11.9%**	**1.05 [0.91, 1.21]**
Total events	71		68			
Heterogeneity: Tau² = 0.00; Chi² = 0.58, df = 2 (P = 0.75); I² = 0%						
Test for overall effect: Z = 0.66 (P = 0.51)						
**Total (95% CI)**		**467**		**455**	**100.0%**	**1.11 [1.06, 1.17]**
Total events	422		365			
Heterogeneity: Tau² = 0.00; Chi² = 9.41, df = 10 (P = 0.49); I² = 0%						
Test for overall effect: Z = 4.07 (P < 0.0001)						
Test for subgroup differences: Chi² = 2.13, df = 2 (P = 0.34), I² = 6.1%						

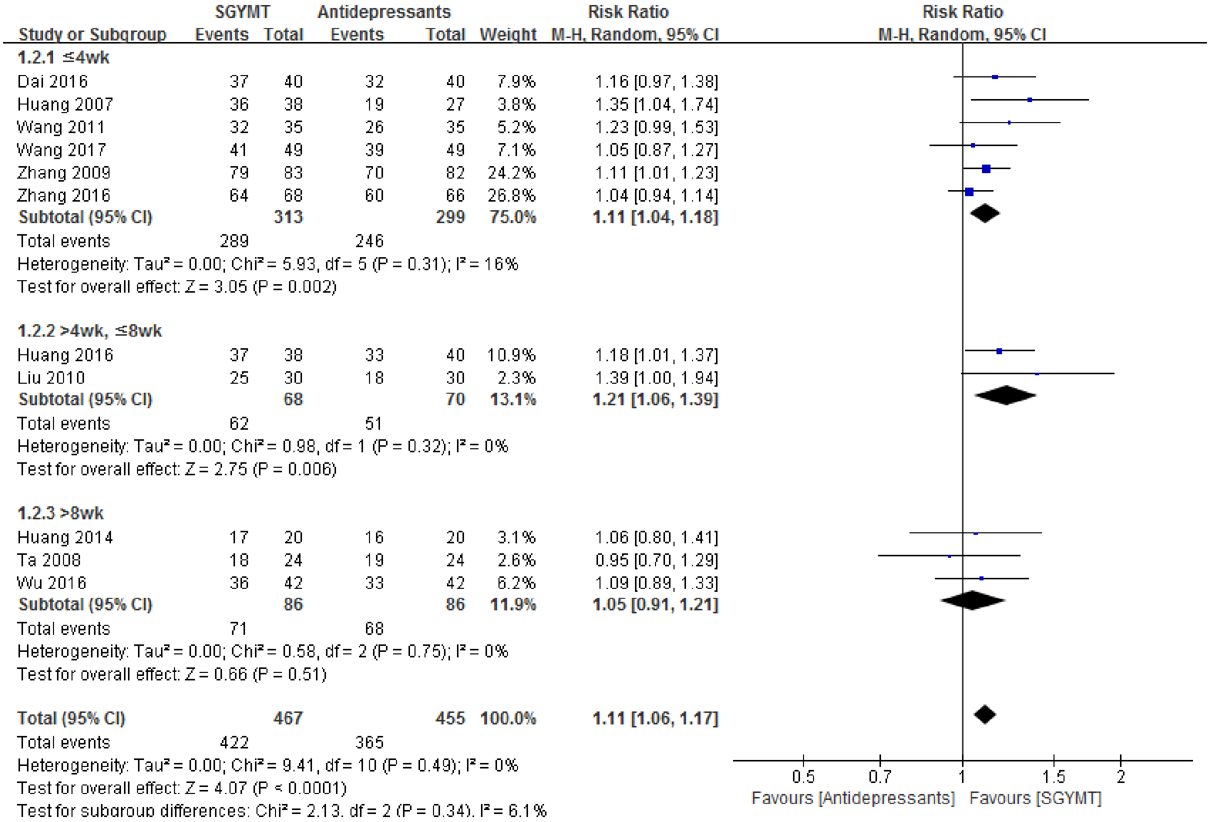

(b) TER based on depression scale. Subgroup analysis according to dosage form

Study or Subgroup	SGYMT Events	SGYMT Total	Antidepressants Events	Antidepressants Total	Weight	Risk Ratio M-H, Random, 95% CI
**3.2.1 decoction**						
Huang 2007	36	38	19	27	3.8%	1.35 [1.04, 1.74]
Huang 2014	17	20	16	20	3.1%	1.06 [0.80, 1.41]
Huang 2016	37	38	33	40	10.9%	1.18 [1.01, 1.37]
Liu 2010	25	30	18	30	2.3%	1.39 [1.00, 1.94]
Ta 2008	18	24	19	24	2.6%	0.95 [0.70, 1.29]
Wang 2011	32	35	26	35	5.2%	1.23 [0.99, 1.53]
Wang 2017	41	49	39	49	7.1%	1.05 [0.87, 1.27]
Wu 2016	36	42	33	42	6.2%	1.09 [0.89, 1.33]
Zhang 2009	79	83	70	82	24.2%	1.11 [1.01, 1.23]
**Subtotal (95% CI)**		**359**		**349**	**65.3%**	**1.14 [1.07, 1.21]**
Total events	321		273			
Heterogeneity: Tau² = 0.00; Chi² = 6.36, df = 8 (P = 0.61); I² = 0%						
Test for overall effect: Z = 4.03 (P < 0.0001)						
**3.2.2 granule**						
Dai 2016	37	40	32	40	7.9%	1.16 [0.97, 1.38]
Zhang 2016	64	68	60	66	26.8%	1.04 [0.94, 1.14]
**Subtotal (95% CI)**		**108**		**106**	**34.7%**	**1.07 [0.96, 1.18]**
Total events	101		92			
Heterogeneity: Tau² = 0.00; Chi² = 1.25, df = 1 (P = 0.26); I² = 20%						
Test for overall effect: Z = 1.26 (P = 0.21)						
**Total (95% CI)**		**467**		**455**	**100.0%**	**1.11 [1.06, 1.17]**
Total events	422		365			
Heterogeneity: Tau² = 0.00; Chi² = 9.41, df = 10 (P = 0.49); I² = 0%						
Test for overall effect: Z = 4.07 (P < 0.0001)						
Test for subgroup differences: Chi² = 1.03, df = 1 (P = 0.31), I² = 2.6%						

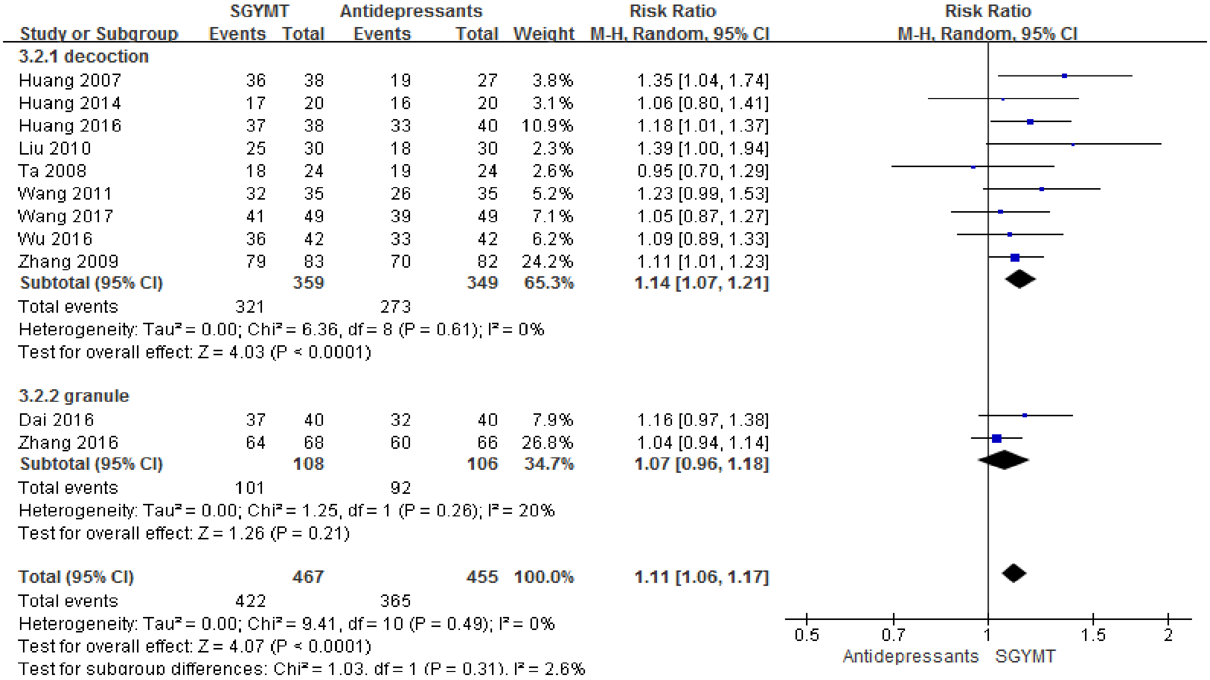

(c) TER based on depression scale. Subgroup analysis according to kinds of antidepressants

Study or Subgroup	SGYMT Events	SGYMT Total	Antidepressants Events	Antidepressants Total	Weight	Risk Ratio M-H, Random, 95% CI
**7.2.1 SSRI**						
Huang 2014	17	20	16	20	3.1%	1.06 [0.80, 1.41]
Liu 2010	25	30	18	30	2.3%	1.39 [1.00, 1.94]
Ta 2008	18	24	19	24	2.6%	0.95 [0.70, 1.29]
Wang 2017	41	49	39	49	7.1%	1.05 [0.87, 1.27]
Wu 2016	36	42	33	42	6.2%	1.09 [0.89, 1.33]
Zhang 2016	64	68	60	66	26.8%	1.04 [0.94, 1.14]
**Subtotal (95% CI)**		**233**		**231**	**48.1%**	**1.06 [0.98, 1.13]**
Total events	201		185			
Heterogeneity: Tau² = 0.00; Chi² = 3.61, df = 5 (P = 0.61); I² = 0%						
Test for overall effect: Z = 1.47 (P = 0.14)						
**7.2.2 TCA**						
Huang 2007	36	38	19	27	3.8%	1.35 [1.04, 1.74]
Wang 2011	32	35	26	35	5.2%	1.23 [0.99, 1.53]
Zhang 2009	79	83	70	82	24.2%	1.11 [1.01, 1.23]
**Subtotal (95% CI)**		**156**		**144**	**33.2%**	**1.17 [1.05, 1.30]**
Total events	147		115			
Heterogeneity: Tau² = 0.00; Chi² = 2.42, df = 2 (P = 0.30); I² = 17%						
Test for overall effect: Z = 2.92 (P = 0.004)						
**7.2.3 Flupentixol/melitracen**						
Dai 2016	37	40	32	40	7.9%	1.16 [0.97, 1.38]
Huang 2016	37	38	33	40	10.9%	1.18 [1.01, 1.37]
**Subtotal (95% CI)**		**78**		**80**	**18.7%**	**1.17 [1.04, 1.31]**
Total events	74		65			
Heterogeneity: Tau² = 0.00; Chi² = 0.03, df = 1 (P = 0.86); I² = 0%						
Test for overall effect: Z = 2.66 (P = 0.008)						
**Total (95% CI)**		**467**		**455**	**100.0%**	**1.11 [1.06, 1.17]**
Total events	422		365			
Heterogeneity: Tau² = 0.00; Chi² = 9.41, df = 10 (P = 0.49); I² = 0%						
Test for overall effect: Z = 4.07 (P < 0.0001)						
Test for subgroup differences: Chi² = 3.62, df = 2 (P = 0.16), I² = 44.7%						

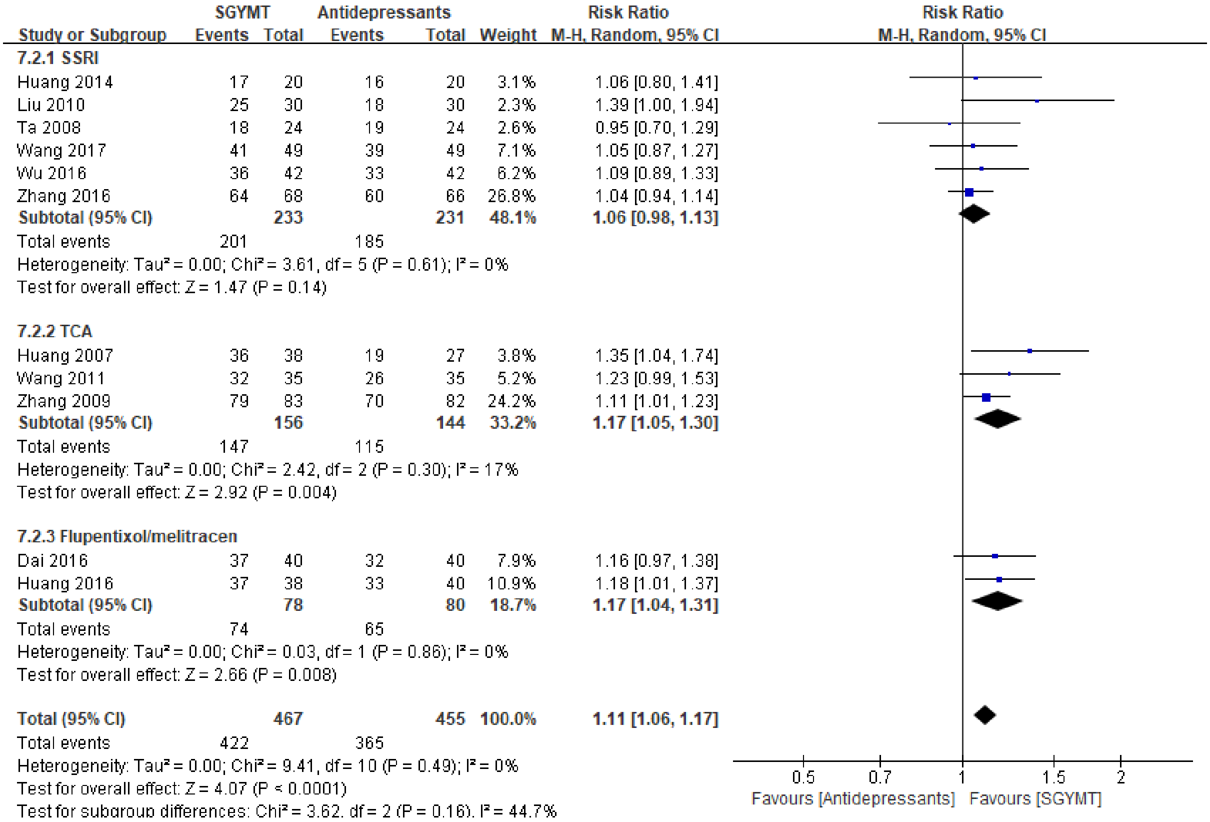

(d) TER based on stroke scale. Subgroup analysis according to treatment period

Study or Subgroup	SGYMT Events	SGYMT Total	Antidepressants Events	Antidepressants Total	Weight	Risk Ratio M-H, Fixed, 95% CI
**1.3.1 ≤4wk**						
Zhang 2016	62	68	57	66	65.9%	1.06 [0.94, 1.19]
**Subtotal (95% CI)**		**68**		**66**	**65.9%**	**1.06 [0.94, 1.19]**
Total events	62		57			
Heterogeneity: Not applicable						
Test for overall effect: Z = 0.88 (P = 0.38)						
**1.3.2 >8wk**						
Huang 2014	17	20	10	20	11.4%	1.70 [1.06, 2.73]
Wu 2016	37	42	20	42	22.8%	1.85 [1.32, 2.59]
**Subtotal (95% CI)**		**62**		**62**	**34.1%**	**1.80 [1.37, 2.37]**
Total events	54		30			
Heterogeneity: Chi² = 0.08, df = 1 (P = 0.78); I² = 0%						
Test for overall effect: Z = 4.20 (P < 0.0001)						
**Total (95% CI)**		**130**		**128**	**100.0%**	**1.31 [1.15, 1.49]**
Total events	116		87			
Heterogeneity: Chi² = 17.40, df = 2 (P = 0.0002); I² = 89%						
Test for overall effect: Z = 4.04 (P < 0.0001)						
Test for subgroup differences: Chi² = 12.15, df = 1 (P = 0.0005), I² = 91.8%						

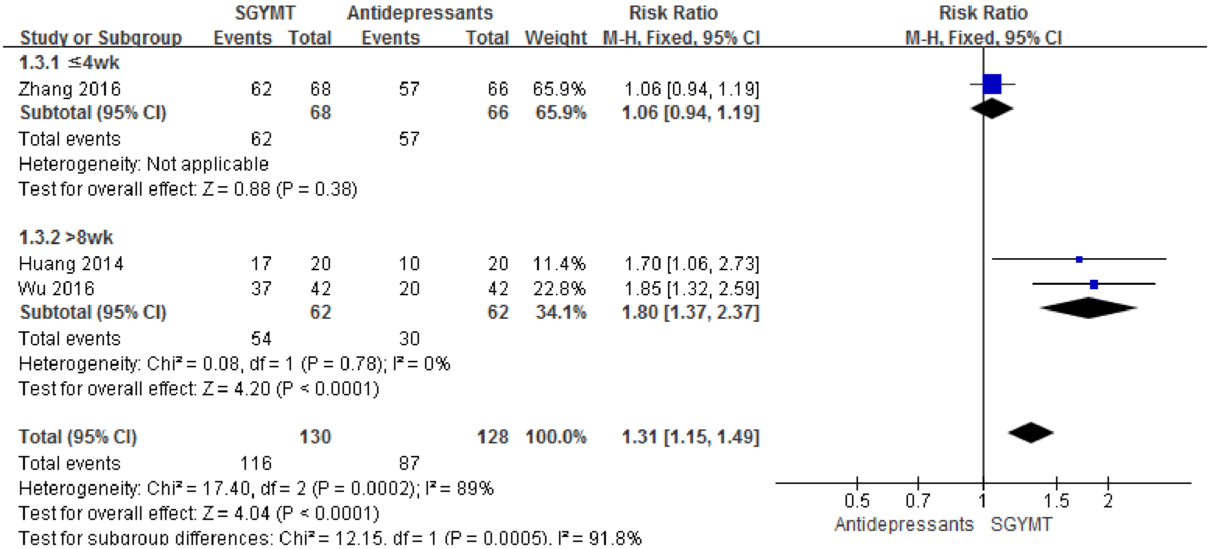

(e) TER based on stroke scale. Subgroup analysis according to dosage form

Study or Subgroup	SGYMT Events	SGYMT Total	Antidepressants Events	Antidepressants Total	Weight	Risk Ratio M-H, Fixed, 95% CI
**3.3.1 decoction**						
Huang 2014	17	20	10	20	11.4%	1.70 [1.06, 2.73]
Wu 2016	37	42	20	42	22.8%	1.85 [1.32, 2.59]
**Subtotal (95% CI)**		**62**		**62**	**34.1%**	**1.80 [1.37, 2.37]**
Total events	54		30			
Heterogeneity: Chi² = 0.08, df = 1 (P = 0.78); I² = 0%						
Test for overall effect: Z = 4.20 (P < 0.0001)						
**3.3.2 granule**						
Zhang 2016	62	68	57	66	65.9%	1.06 [0.94, 1.19]
**Subtotal (95% CI)**		**68**		**66**	**65.9%**	**1.06 [0.94, 1.19]**
Total events	62		57			
Heterogeneity: Not applicable						
Test for overall effect: Z = 0.88 (P = 0.38)						
**Total (95% CI)**		**130**		**128**	**100.0%**	**1.31 [1.15, 1.49]**
Total events	116		87			
Heterogeneity: Chi² = 17.40, df = 2 (P = 0.0002); I² = 89%						
Test for overall effect: Z = 4.04 (P < 0.0001)						
Test for subgroup differences: Chi² = 12.15, df = 1 (P = 0.0005), I² = 91.8%						

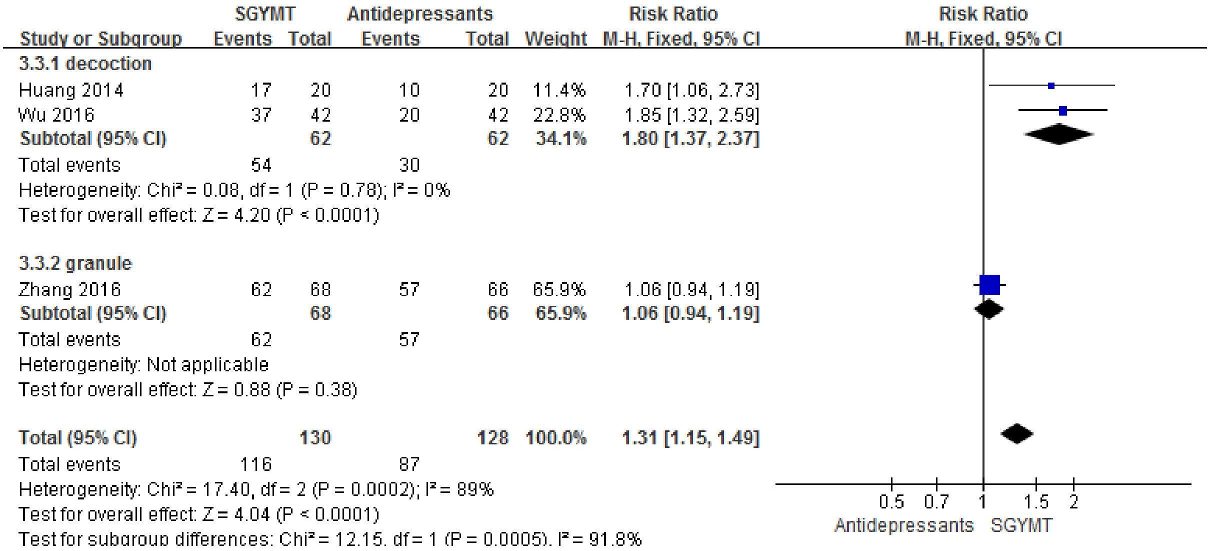

(f) NIHSS score. Subgroup analysis according to treatment period

Study or Subgroup	SGYMT Mean	SGYMT SD	SGYMT Total	Antidepressants Mean	Antidepressants SD	Antidepressants Total	Weight	Mean Difference IV, Fixed, 95% CI
**1.4.1 ≤4wk**								
Liu 2015a	4.84	1.81	28	5.21	2.14	32	30.7%	-0.37 [-1.37, 0.63]
**Subtotal (95% CI)**			**28**			**32**	**30.7%**	**-0.37 [-1.37, 0.63]**
Heterogeneity: Not applicable								
Test for overall effect: Z = 0.73 (P = 0.47)								
**1.4.2 >4wk, ≤8wk**								
Huang 2016	8.64	1.25	38	9.69	1.72	40	69.3%	-1.05 [-1.71, -0.39]
**Subtotal (95% CI)**			**38**			**40**	**69.3%**	**-1.05 [-1.71, -0.39]**
Heterogeneity: Not applicable								
Test for overall effect: Z = 3.10 (P = 0.002)								
**Total (95% CI)**			**66**			**72**	**100.0%**	**-0.84 [-1.40, -0.29]**
Heterogeneity: Chi² = 1.23, df = 1 (P = 0.27); I² = 19%								
Test for overall effect: Z = 2.98 (P = 0.003)								
Test for subgroup differences: Chi² = 1.23, df = 1 (P = 0.27), I² = 18.9%								

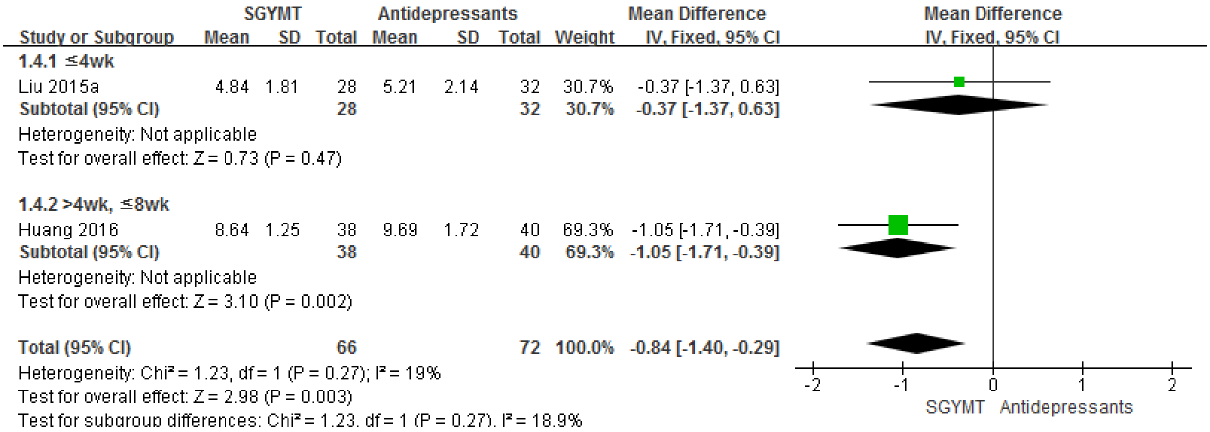

(g) NIHSS score. Subgroup analysis according to kinds of antidepressants

Study or Subgroup	SGYMT Mean	SGYMT SD	SGYMT Total	Antidepressants Mean	Antidepressants SD	Antidepressants Total	Weight	Mean Difference IV, Fixed, 95% CI
**7.4.1 SSRI**								
Liu 2015a	4.84	1.81	28	5.21	2.14	32	30.7%	-0.37 [-1.37, 0.63]
**Subtotal (95% CI)**			**28**			**32**	**30.7%**	**-0.37 [-1.37, 0.63]**
Heterogeneity: Not applicable								
Test for overall effect: Z = 0.73 (P = 0.47)								
**7.4.2 Flupentixol/melitracen**								
Huang 2016	8.64	1.25	38	9.69	1.72	40	69.3%	-1.05 [-1.71, -0.39]
**Subtotal (95% CI)**			**38**			**40**	**69.3%**	**-1.05 [-1.71, -0.39]**
Heterogeneity: Not applicable								
Test for overall effect: Z = 3.10 (P = 0.002)								
**Total (95% CI)**			**66**			**72**	**100.0%**	**-0.84 [-1.40, -0.29]**
Heterogeneity: Chi² = 1.23, df = 1 (P = 0.27); I² = 19%								
Test for overall effect: Z = 2.98 (P = 0.003)								
Test for subgroup differences: Chi² = 1.23, df = 1 (P = 0.27), I² = 18.9%								

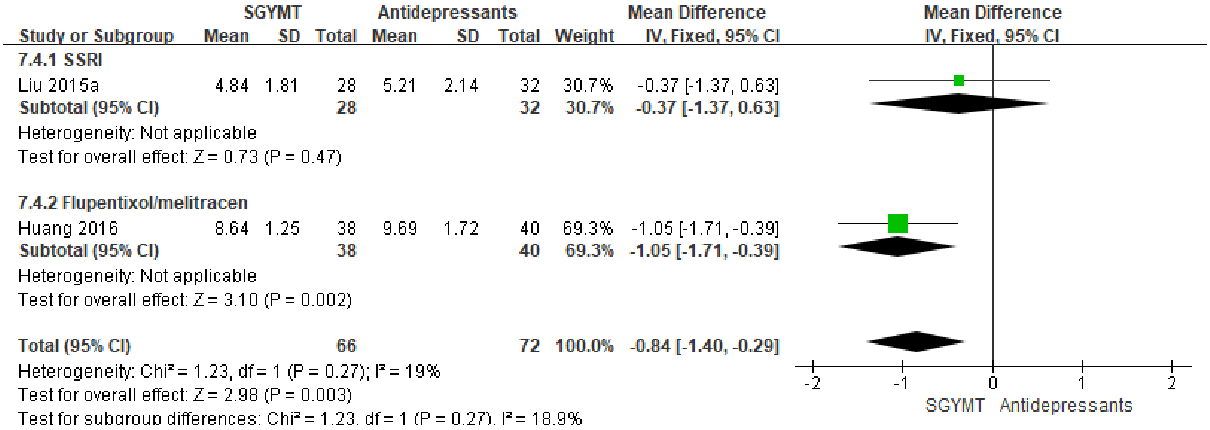

(h) CSS score. Subgroup analysis according to treatment period

Study or Subgroup	SGYMT Mean	SGYMT SD	SGYMT Total	Antidepressants Mean	Antidepressants SD	Antidepressants Total	Weight	Mean Difference IV, Fixed, 95% CI
**1.5.1 >4wk, ≤8wk**								
Liu 2010	9.4	3.5	30	13.6	3.4	30	48.9%	-4.20 [-5.95, -2.45]
**Subtotal (95% CI)**			**30**			**30**	**48.9%**	**-4.20 [-5.95, -2.45]**
Heterogeneity: Not applicable								
Test for overall effect: Z = 4.71 (P < 0.00001)								
**1.5.2 >8wk**								
Huang 2014	11.42	4.47	20	18.25	4.27	20	20.3%	-6.83 [-9.54, -4.12]
Wu 2016	12.66	5.06	42	18.94	5.23	42	30.8%	-6.28 [-8.48, -4.08]
**Subtotal (95% CI)**			**62**			**62**	**51.1%**	**-6.50 [-8.21, -4.79]**
Heterogeneity: Chi² = 0.10, df = 1 (P = 0.76); I² = 0%								
Test for overall effect: Z = 7.46 (P < 0.00001)								
**Total (95% CI)**			**92**			**92**	**100.0%**	**-5.37 [-6.60, -4.15]**
Heterogeneity: Chi² = 3.50, df = 2 (P = 0.17); I² = 43%								
Test for overall effect: Z = 8.63 (P < 0.00001)								
Test for subgroup differences: Chi² = 3.40, df = 1 (P = 0.07), I² = 70.6%								

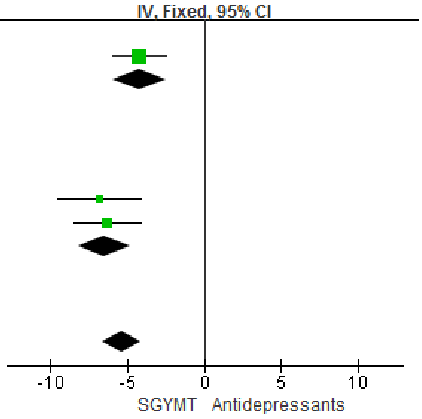

(i) Barthel index. Subgroup analysis according to treatment period

Study or Subgroup	SGYMT Mean	SGYMT SD	SGYMT Total	Antidepressants Mean	Antidepressants SD	Antidepressants Total	Weight	Mean Difference IV, Fixed, 95% CI
**1.7.1 ≤4wk**								
Liu 2015a	86.65	8.15	28	85.91	9.03	32	27.1%	0.74 [-3.61, 5.09]
Zhang 2016	73.56	8.891	68	69.17	9.429	66	53.2%	4.39 [1.29, 7.49]
**Subtotal (95% CI)**			**96**			**98**	**80.4%**	**3.16 [0.63, 5.68]**
Heterogeneity: Chi² = 1.79, df = 1 (P = 0.18); I² = 44%								
Test for overall effect: Z = 2.45 (P = 0.01)								
**1.7.2 >8wk**								
Huang 2014	72.86	8.14	20	63.87	8.35	20	19.6%	8.99 [3.88, 14.10]
**Subtotal (95% CI)**			**20**			**20**	**19.6%**	**8.99 [3.88, 14.10]**
Heterogeneity: Not applicable								
Test for overall effect: Z = 3.45 (P = 0.0006)								
**Total (95% CI)**			**116**			**118**	**100.0%**	**4.30 [2.04, 6.57]**
Heterogeneity: Chi² = 5.81, df = 2 (P = 0.05); I² = 66%								
Test for overall effect: Z = 3.72 (P = 0.0002)								
Test for subgroup differences: Chi² = 4.02, df = 1 (P = 0.04), I² = 75.1%								

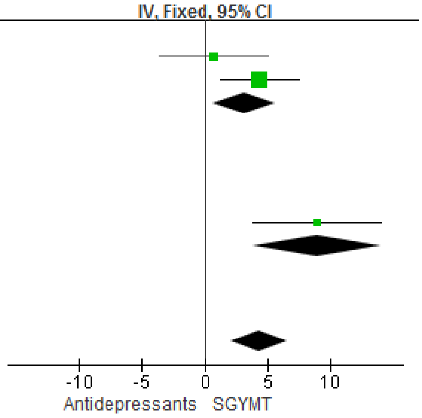

(j) Barthel index. Subgroup analysis according to dosage form

Study or Subgroup	SGYMT Mean	SGYMT SD	SGYMT Total	Antidepressants Mean	Antidepressants SD	Antidepressants Total	Weight	Mean Difference IV, Fixed, 95% CI
**3.7.1 decoction**								
Huang 2014	72.86	8.14	20	63.87	8.35	20	19.6%	8.99 [3.88, 14.10]
Liu 2015a	86.65	8.15	28	85.91	9.03	32	27.1%	0.74 [-3.61, 5.09]
**Subtotal (95% CI)**			**48**			**52**	**46.8%**	**4.20 [0.89, 7.52]**
Heterogeneity: Chi² = 5.81, df = 1 (P = 0.02); I² = 83%								
Test for overall effect: Z = 2.49 (P = 0.01)								
**3.7.2 granule**								
Zhang 2016	73.56	8.891	68	69.17	9.429	66	53.2%	4.39 [1.29, 7.49]
**Subtotal (95% CI)**			**68**			**66**	**53.2%**	**4.39 [1.29, 7.49]**
Heterogeneity: Not applicable								
Test for overall effect: Z = 2.77 (P = 0.006)								
**Total (95% CI)**			**116**			**118**	**100.0%**	**4.30 [2.04, 6.57]**
Heterogeneity: Chi² = 5.81, df = 2 (P = 0.05); I² = 66%								
Test for overall effect: Z = 3.72 (P = 0.0002)								
Test for subgroup differences: Chi² = 0.01, df = 1 (P = 0.94), I² = 0%								

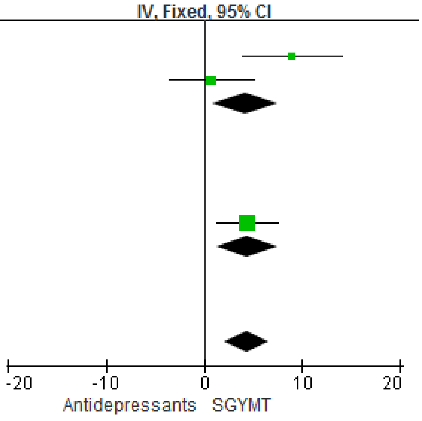

Supplemental digital content 6. Forest plots for other outcomes in comparison of SGYMT plus antidepressants versus antidepressants alone

(a) TER based on depression scale. Subgroup analysis according to treatment period

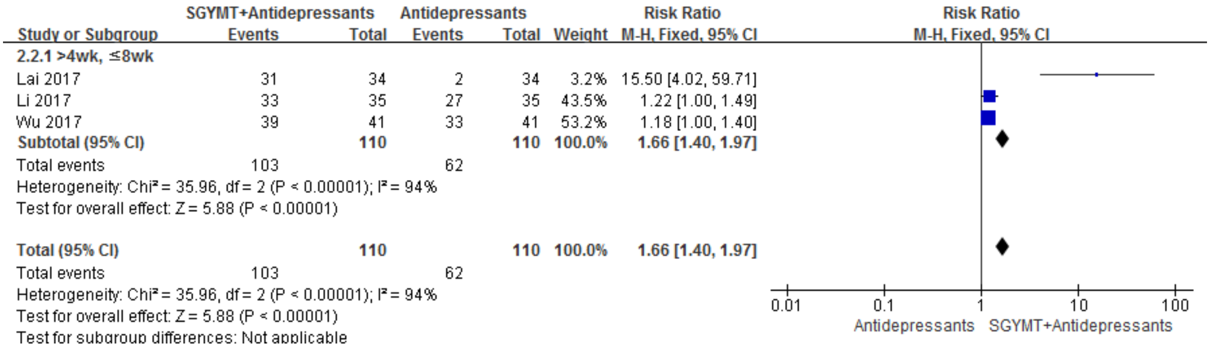

(b) TER based on depression scale. Subgroup analysis according to kinds of antidepressants

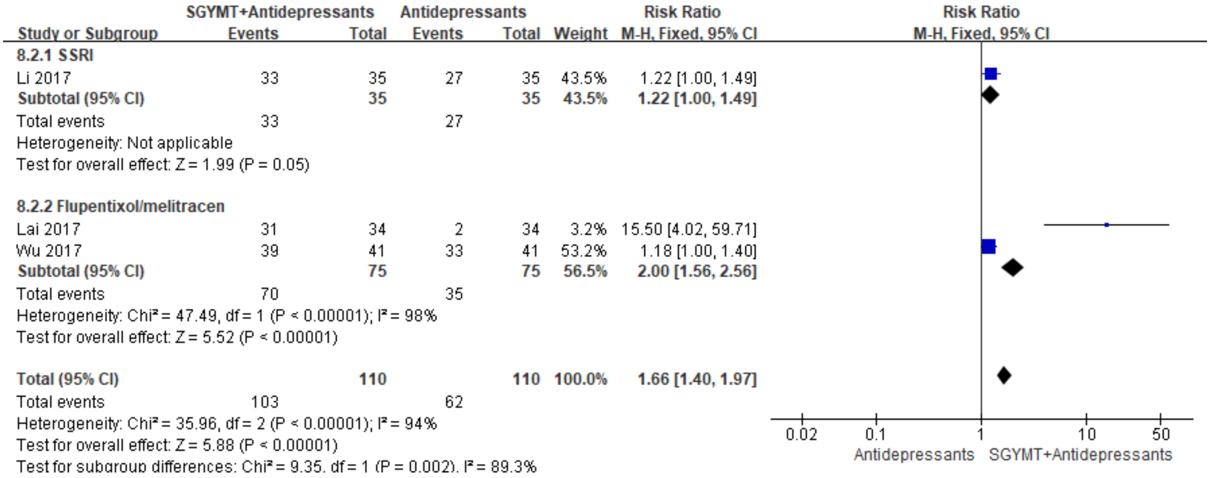

(c) NIHSS score. Subgroup analysis according to treatment period

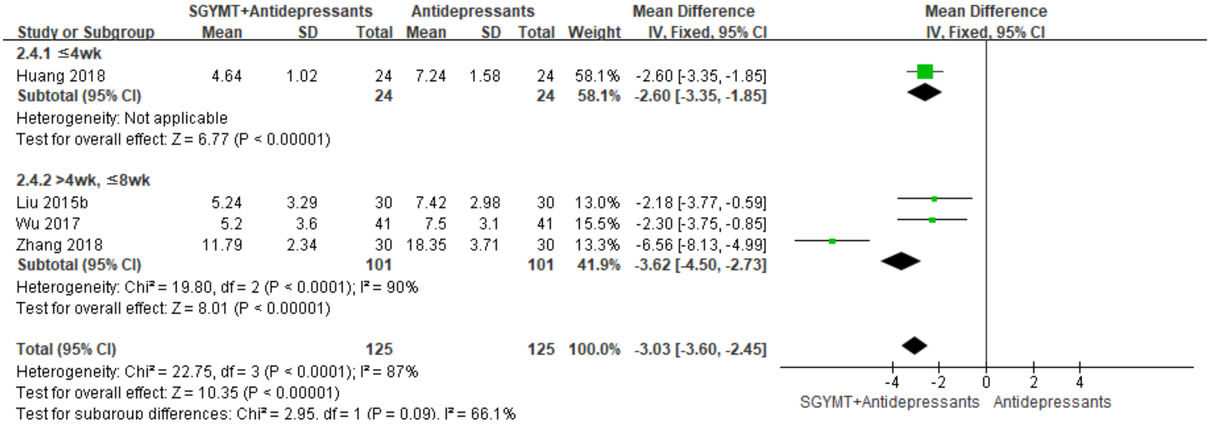

(d) NIHSS score. Subgroup analysis according to kinds of antidepressants

	SGYMT+Antidepressants			Antidepressants				Mean Difference
Study or Subgroup	Mean	SD	Total	Mean	SD	Total	Weight	IV, Fixed, 95% CI
**8.4.1 SSRI**								
Zhang 2018	11.79	2.34	30	18.35	3.71	30	13.3%	-6.56 [-8.13, -4.99]
**Subtotal (95% CI)**			**30**			**30**	**13.3%**	**-6.56 [-8.13, -4.99]**
Heterogeneity: Not applicable								
Test for overall effect: Z = 8.19 (P < 0.00001)								
**8.4.2 Flupentixol/melitracen**								
Huang 2018	4.64	1.02	24	7.24	1.58	24	58.1%	-2.60 [-3.35, -1.85]
Liu 2015b	5.24	3.29	30	7.42	2.98	30	13.0%	-2.18 [-3.77, -0.59]
Wu 2017	5.2	3.6	41	7.5	3.1	41	15.5%	-2.30 [-3.75, -0.85]
**Subtotal (95% CI)**			**95**			**95**	**86.7%**	**-2.48 [-3.10, -1.87]**
Heterogeneity: $Chi^2 = 0.29$, df = 2 (P = 0.86); $I^2 = 0\%$								
Test for overall effect: Z = 7.90 (P < 0.00001)								
**Total (95% CI)**			**125**			**125**	**100.0%**	**-3.03 [-3.60, -2.45]**
Heterogeneity: $Chi^2 = 22.75$, df = 3 (P < 0.0001); $I^2 = 87\%$								
Test for overall effect: Z = 10.35 (P < 0.00001)								
Test for subgroup differences: $Chi^2 = 22.46$, df = 1 (P < 0.00001), $I^2 = 95.5\%$								

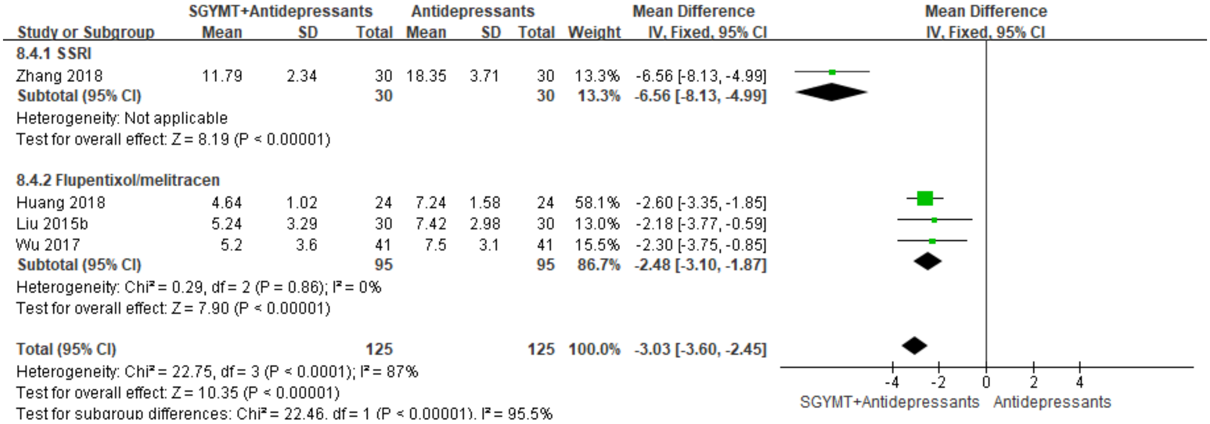

Supplemental digital content 7. Results of meta-analysis analyzed using pre-registered fixed-effects model

(a)HAMD scores (SGYMT versus Antidepressants). Subgroup analysis according to treatment period

	SGYMT			Antidepressants				Mean Difference
Study or Subgroup	Mean	SD	Total	Mean	SD	Total	Weight	IV, Fixed, 95% CI
**1.1.1 ≤4wk**								
Dai 2016	7.02	3.15	40	10.28	3.74	40	7.6%	-3.26 [-4.78, -1.74]
Liu 2015a	6.21	2.19	28	7.98	1.15	32	21.2%	-1.77 [-2.67, -0.87]
Wang 2017	13.62	4.17	49	14.55	4.08	49	6.5%	-0.93 [-2.56, 0.70]
**Subtotal (95% CI)**			**117**			**121**	**35.3%**	**-1.93 [-2.64, -1.23]**
Heterogeneity: Chi² = 4.52, df = 2 (P = 0.10); I² = 56%								
Test for overall effect: Z = 5.41 (P < 0.00001)								
**1.1.2 >4wk, ≤8wk**								
Huang 2016	15.28	2.71	38	17.72	3.03	40	10.7%	-2.44 [-3.71, -1.17]
Liu 2010	9.6	2.2	30	12.3	2.4	30	12.8%	-2.70 [-3.87, -1.53]
Zhang 2011	7.06	0.85	30	9.47	1.84	30	33.0%	-2.41 [-3.14, -1.68]
**Subtotal (95% CI)**			**98**			**100**	**56.4%**	**-2.48 [-3.04, -1.93]**
Heterogeneity: Chi² = 0.18, df = 2 (P = 0.92); I² = 0%								
Test for overall effect: Z = 8.77 (P < 0.00001)								
**1.1.3 >8wk**								
Huang 2014	13.85	3.99	20	14.35	3.89	20	2.9%	-0.50 [-2.94, 1.94]
Wu 2016	14.49	4.22	42	15.24	4.15	42	5.4%	-0.75 [-2.54, 1.04]
**Subtotal (95% CI)**			**62**			**62**	**8.3%**	**-0.66 [-2.11, 0.78]**
Heterogeneity: Chi² = 0.03, df = 1 (P = 0.87); I² = 0%								
Test for overall effect: Z = 0.90 (P = 0.37)								
**Total (95% CI)**			**277**			**283**	**100.0%**	**-2.14 [-2.55, -1.72]**
Heterogeneity: Chi² = 10.53, df = 7 (P = 0.16); I² = 34%								
Test for overall effect: Z = 10.06 (P < 0.00001)								
Test for subgroup differences: Chi² = 5.81, df = 2 (P = 0.05), I² = 65.6%								

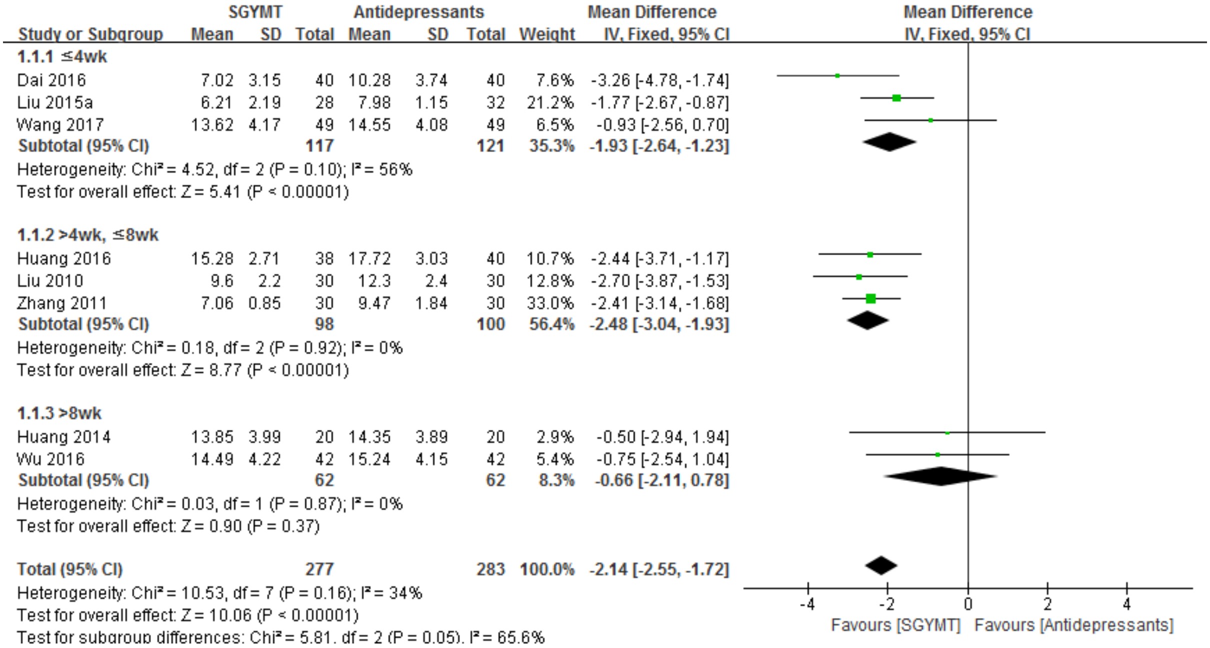

(b)HAMD scores (SGYMT versus Antidepressants). Subgroup analysis according to dosage form

	SGYMT			Antidepressants				Mean Difference
Study or Subgroup	Mean	SD	Total	Mean	SD	Total	Weight	IV, Fixed, 95% CI
**3.1.1 decoction**								
Huang 2014	13.85	3.99	20	14.35	3.89	20	2.9%	-0.50 [-2.94, 1.94]
Huang 2016	15.28	2.71	38	17.72	3.03	40	10.7%	-2.44 [-3.71, -1.17]
Liu 2010	9.6	2.2	30	12.3	2.4	30	12.8%	-2.70 [-3.87, -1.53]
Liu 2015a	6.21	2.19	28	7.98	1.15	32	21.2%	-1.77 [-2.67, -0.87]
Wang 2017	13.62	4.17	49	14.55	4.08	49	6.5%	-0.93 [-2.56, 0.70]
Wu 2016	14.49	4.22	42	15.24	4.15	42	5.4%	-0.75 [-2.54, 1.04]
Zhang 2011	7.06	0.85	30	9.47	1.84	30	33.0%	-2.41 [-3.14, -1.68]
**Subtotal (95% CI)**			**237**			**243**	**92.4%**	**-2.05 [-2.48, -1.61]**
Heterogeneity: Chi² = 8.25, df = 6 (P = 0.22); I² = 27%								
Test for overall effect: Z = 9.26 (P < 0.00001)								
**3.1.2 granule**								
Dai 2016	7.02	3.15	40	10.28	3.74	40	7.6%	-3.26 [-4.78, -1.74]
**Subtotal (95% CI)**			**40**			**40**	**7.6%**	**-3.26 [-4.78, -1.74]**
Heterogeneity: Not applicable								
Test for overall effect: Z = 4.22 (P < 0.0001)								
**Total (95% CI)**			**277**			**283**	**100.0%**	**-2.14 [-2.55, -1.72]**
Heterogeneity: Chi² = 10.53, df = 7 (P = 0.16); I² = 34%								
Test for overall effect: Z = 10.06 (P < 0.00001)								
Test for subgroup differences: Chi² = 2.28, df = 1 (P = 0.13), I² = 56.2%								

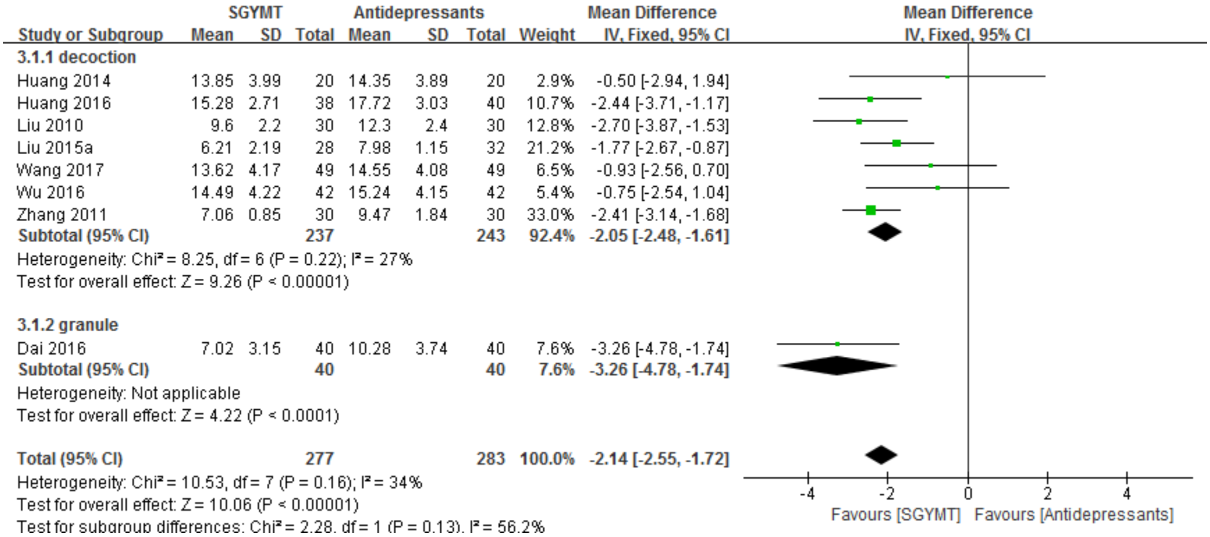

(c)HAMD scores (SGYMT versus Antidepressants). Subgroup analysis according to kinds of antidepressants

Study or Subgroup	SGYMT Mean	SGYMT SD	SGYMT Total	Antidepressants Mean	Antidepressants SD	Antidepressants Total	Weight	Mean Difference IV, Fixed, 95% CI
**7.1.1 SSRI**								
Huang 2014	13.85	3.99	20	14.35	3.89	20	2.9%	-0.50 [-2.94, 1.94]
Liu 2010	9.6	2.2	30	12.3	2.4	30	12.8%	-2.70 [-3.87, -1.53]
Liu 2015a	6.21	2.19	28	7.98	1.15	32	21.2%	-1.77 [-2.67, -0.87]
Wang 2017	13.62	4.17	49	14.55	4.08	49	6.5%	-0.93 [-2.56, 0.70]
Wu 2016	14.49	4.22	42	15.24	4.15	42	5.4%	-0.75 [-2.54, 1.04]
**Subtotal (95% CI)**			**169**			**173**	**48.8%**	**-1.71 [-2.31, -1.12]**
Heterogeneity: Chi² = 5.71, df = 4 (P = 0.22); I² = 30%								
Test for overall effect: Z = 5.63 (P < 0.00001)								
**7.1.2 Flupentixol/melitracen**								
Dai 2016	7.02	3.15	40	10.28	3.74	40	7.6%	-3.26 [-4.78, -1.74]
Huang 2016	15.28	2.71	38	17.72	3.03	40	10.7%	-2.44 [-3.71, -1.17]
Zhang 2011	7.06	0.85	30	9.47	1.84	30	33.0%	-2.41 [-3.14, -1.68]
**Subtotal (95% CI)**			**108**			**110**	**51.2%**	**-2.54 [-3.12, -1.96]**
Heterogeneity: Chi² = 1.01, df = 2 (P = 0.60); I² = 0%								
Test for overall effect: Z = 8.56 (P < 0.00001)								
**Total (95% CI)**			**277**			**283**	**100.0%**	**-2.14 [-2.55, -1.72]**
Heterogeneity: Chi² = 10.53, df = 7 (P = 0.16); I² = 34%								
Test for overall effect: Z = 10.06 (P < 0.00001)								
Test for subgroup differences: Chi² = 3.80, df = 1 (P = 0.05), I² = 73.7%								

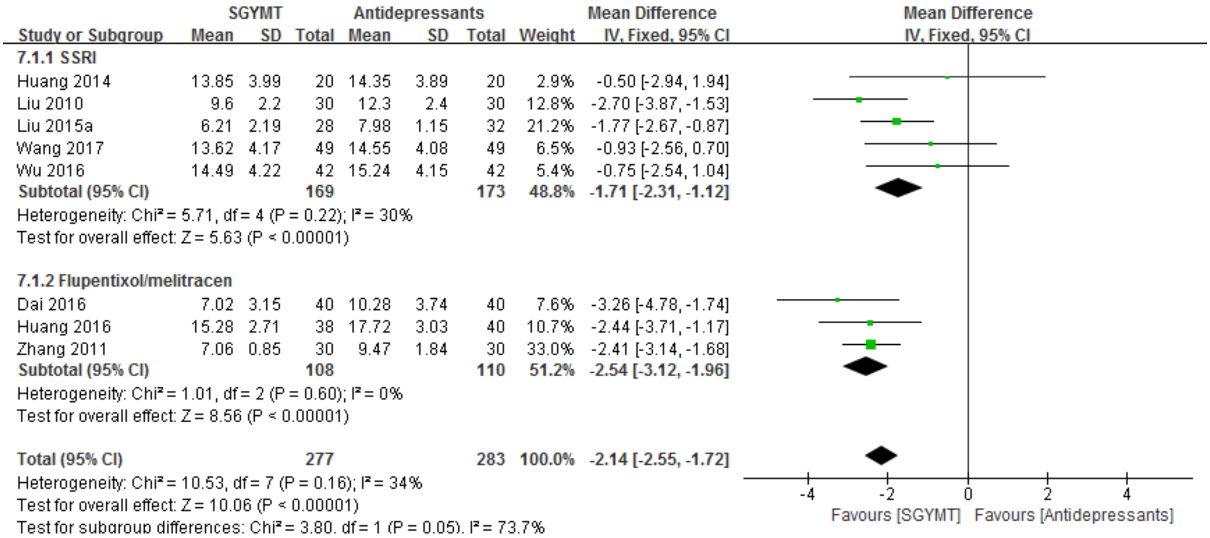

(d)TER based on depression scale (SGYMT versus Antidepressants). Subgroup analysis according to treatment period

Study or Subgroup	SGYMT Events	SGYMT Total	Antidepressants Events	Antidepressants Total	Weight	Risk Ratio M-H, Fixed, 95% CI
**1.2.1 ≤4wk**						
Dai 2016	37	40	32	40	8.7%	1.16 [0.97, 1.38]
Huang 2007	36	38	19	27	6.0%	1.35 [1.04, 1.74]
Wang 2011	32	35	26	35	7.1%	1.23 [0.99, 1.53]
Wang 2017	41	49	39	49	10.6%	1.05 [0.87, 1.27]
Zhang 2009	79	83	70	82	19.1%	1.11 [1.01, 1.23]
Zhang 2016	64	68	60	66	16.5%	1.04 [0.94, 1.14]
**Subtotal (95% CI)**		**313**		**299**	**68.0%**	**1.12 [1.06, 1.20]**
Total events	289		246			
Heterogeneity: Chi² = 5.93, df = 5 (P = 0.31); I² = 16%						
Test for overall effect: Z = 3.68 (P = 0.0002)						
**1.2.2 >4wk, ≤8wk**						
Huang 2016	37	38	33	40	8.7%	1.18 [1.01, 1.37]
Liu 2010	25	30	18	30	4.9%	1.39 [1.00, 1.94]
**Subtotal (95% CI)**		**68**		**70**	**13.6%**	**1.26 [1.07, 1.47]**
Total events	62		51			
Heterogeneity: Chi² = 0.98, df = 1 (P = 0.32); I² = 0%						
Test for overall effect: Z = 2.83 (P = 0.005)						
**1.2.3 >8wk**						
Huang 2014	17	20	16	20	4.3%	1.06 [0.80, 1.41]
Ta 2008	18	24	19	24	5.2%	0.95 [0.70, 1.29]
Wu 2016	36	42	33	42	9.0%	1.09 [0.89, 1.33]
**Subtotal (95% CI)**		**86**		**86**	**18.4%**	**1.04 [0.90, 1.21]**
Total events	71		68			
Heterogeneity: Chi² = 0.58, df = 2 (P = 0.75); I² = 0%						
Test for overall effect: Z = 0.58 (P = 0.56)						
**Total (95% CI)**		**467**		**455**	**100.0%**	**1.13 [1.07, 1.19]**
Total events	422		365			
Heterogeneity: Chi² = 9.41, df = 10 (P = 0.49); I² = 0%						
Test for overall effect: Z = 4.31 (P < 0.0001)						
Test for subgroup differences: Chi² = 2.86, df = 2 (P = 0.24), I² = 30.1%						

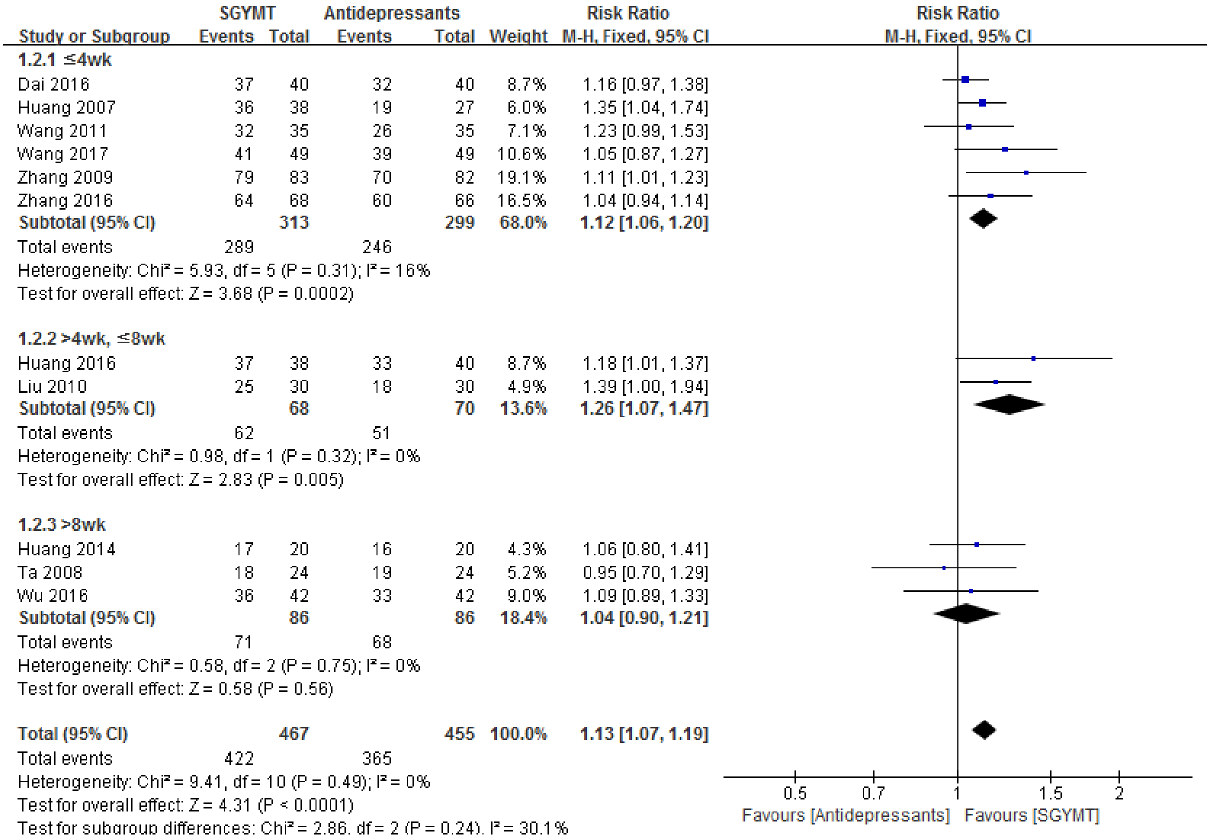

(e) TER based on depression scale (SGYMT versus Antidepressants). Subgroup analysis according to dosage form

Study or Subgroup	SGYMT Events	SGYMT Total	Antidepressants Events	Antidepressants Total	Weight	Risk Ratio M-H, Fixed, 95% CI
**3.2.1 decoction**						
Huang 2007	36	38	19	27	6.0%	1.35 [1.04, 1.74]
Huang 2014	17	20	16	20	4.3%	1.06 [0.80, 1.41]
Huang 2016	37	38	33	40	8.7%	1.18 [1.01, 1.37]
Liu 2010	25	30	18	30	4.9%	1.39 [1.00, 1.94]
Ta 2008	18	24	19	24	5.2%	0.95 [0.70, 1.29]
Wang 2011	32	35	26	35	7.1%	1.23 [0.99, 1.53]
Wang 2017	41	49	39	49	10.6%	1.05 [0.87, 1.27]
Wu 2016	36	42	33	42	9.0%	1.09 [0.89, 1.33]
Zhang 2009	79	83	70	82	19.1%	1.11 [1.01, 1.23]
**Subtotal (95% CI)**		**359**		**349**	**74.8%**	**1.14 [1.07, 1.22]**
Total events	321		273			
Heterogeneity: Chi² = 6.36, df = 8 (P = 0.61); I² = 0%						
Test for overall effect: Z = 3.98 (P < 0.0001)						
**3.2.2 granule**						
Dai 2016	37	40	32	40	8.7%	1.16 [0.97, 1.38]
Zhang 2016	64	68	60	66	16.5%	1.04 [0.94, 1.14]
**Subtotal (95% CI)**		**108**		**106**	**25.2%**	**1.08 [0.99, 1.18]**
Total events	101		92			
Heterogeneity: Chi² = 1.25, df = 1 (P = 0.26); I² = 20%						
Test for overall effect: Z = 1.63 (P = 0.10)						
**Total (95% CI)**		**467**		**455**	**100.0%**	**1.13 [1.07, 1.19]**
Total events	422		365			
Heterogeneity: Chi² = 9.41, df = 10 (P = 0.49); I² = 0%						
Test for overall effect: Z = 4.31 (P < 0.0001)						
Test for subgroup differences: Chi² = 1.13, df = 1 (P = 0.29), I² = 11.2%						

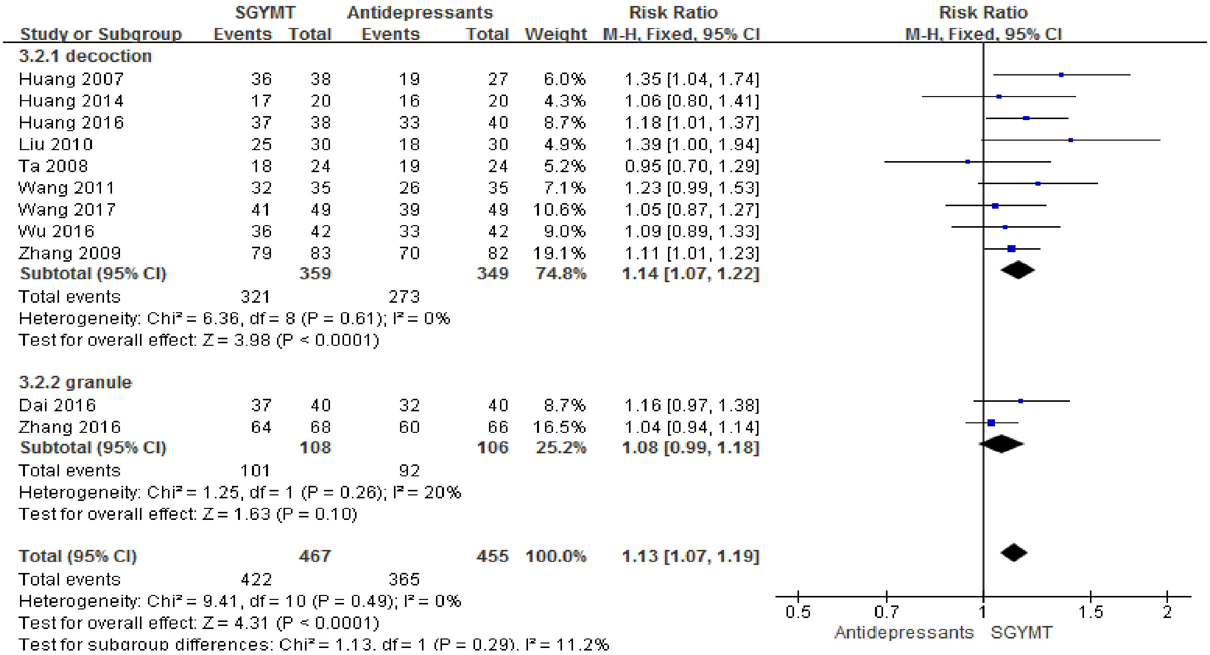

(f) TER based on depression scale (SGYMT versus Antidepressants). Subgroup analysis according to kinds of antidepressants

Study or Subgroup	SGYMT Events	SGYMT Total	Antidepressants Events	Antidepressants Total	Weight	Risk Ratio M-H, Fixed, 95% CI
**7.2.1 SSRI**						
Huang 2014	17	20	16	20	4.3%	1.06 [0.80, 1.41]
Liu 2010	25	30	18	30	4.9%	1.39 [1.00, 1.94]
Ta 2008	18	24	19	24	5.2%	0.95 [0.70, 1.29]
Wang 2017	41	49	39	49	10.6%	1.05 [0.87, 1.27]
Wu 2016	36	42	33	42	9.0%	1.09 [0.89, 1.33]
Zhang 2016	64	68	60	66	16.5%	1.04 [0.94, 1.14]
**Subtotal (95% CI)**		**233**		**231**	**50.4%**	**1.08 [0.99, 1.17]**
Total events	201		185			
Heterogeneity: Chi² = 3.61, df = 5 (P = 0.61); I² = 0%						
Test for overall effect: Z = 1.77 (P = 0.08)						
**7.2.2 TCA**						
Huang 2007	36	38	19	27	6.0%	1.35 [1.04, 1.74]
Wang 2011	32	35	26	35	7.1%	1.23 [0.99, 1.53]
Zhang 2009	79	83	70	82	19.1%	1.11 [1.01, 1.23]
**Subtotal (95% CI)**		**156**		**144**	**32.2%**	**1.18 [1.08, 1.30]**
Total events	147		115			
Heterogeneity: Chi² = 2.42, df = 2 (P = 0.30); I² = 17%						
Test for overall effect: Z = 3.59 (P = 0.0003)						
**7.2.3 Flupentixol/melitracen**						
Dai 2016	37	40	32	40	8.7%	1.16 [0.97, 1.38]
Huang 2016	37	38	33	40	8.7%	1.18 [1.01, 1.37]
**Subtotal (95% CI)**		**78**		**80**	**17.4%**	**1.17 [1.04, 1.31]**
Total events	74		65			
Heterogeneity: Chi² = 0.03, df = 1 (P = 0.86); I² = 0%						
Test for overall effect: Z = 2.60 (P = 0.009)						
**Total (95% CI)**		**467**		**455**	**100.0%**	**1.13 [1.07, 1.19]**
Total events	422		365			
Heterogeneity: Chi² = 9.41, df = 10 (P = 0.49); I² = 0%						
Test for overall effect: Z = 4.31 (P < 0.0001)						
Test for subgroup differences: Chi² = 2.68, df = 2 (P = 0.26), I² = 25.3%						

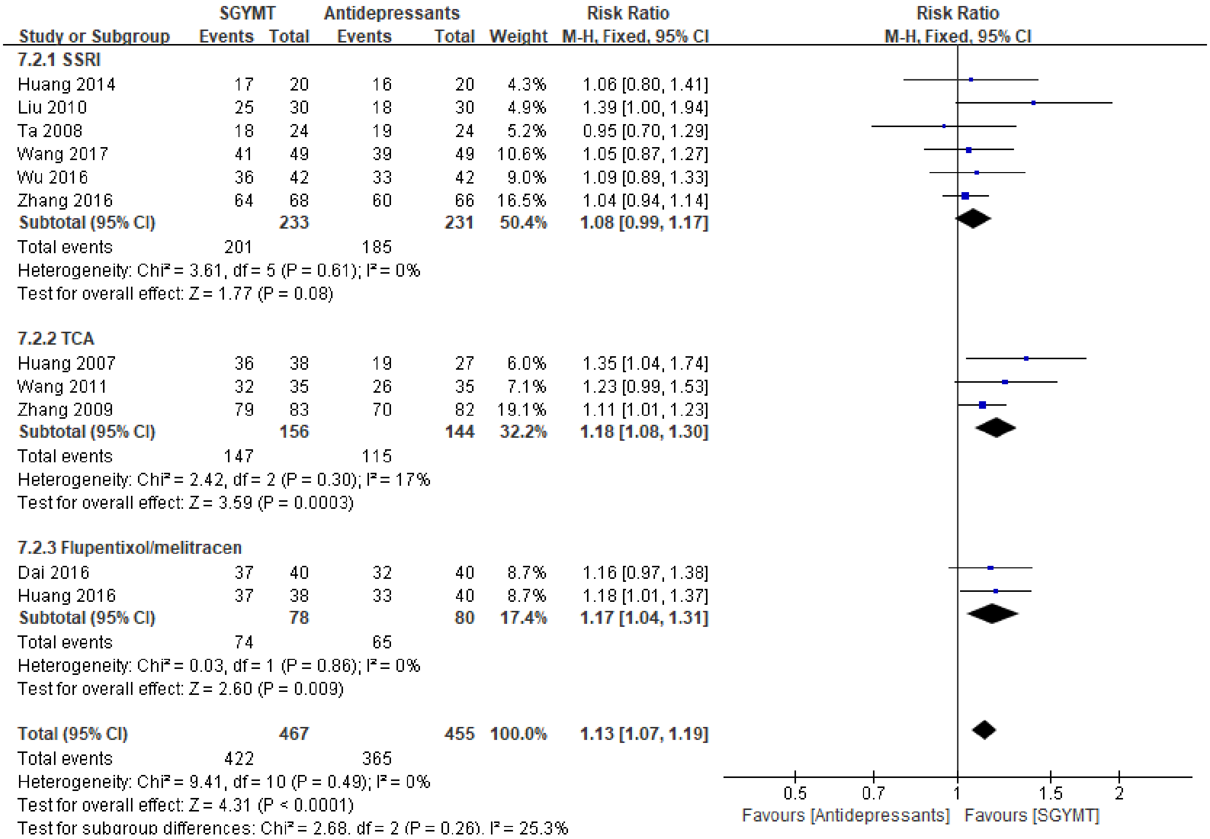

(g) Incidence of adverse events (SGYMT versus Antidepressants). Subgroup analysis according to treatment period

Study or Subgroup	SGYMT Events	SGYMT Total	Antidepressants Events	Antidepressants Total	Weight	Risk Ratio M-H, Fixed, 95% CI
**1.9.1 ≤4wk**						
Dai 2016	0	40	7	40	17.4%	0.07 [0.00, 1.13]
Wang 2017	0	49	3	49	8.1%	0.14 [0.01, 2.69]
**Subtotal (95% CI)**		**89**		**89**	**25.6%**	**0.09 [0.01, 0.69]**
Total events	0		10			
Heterogeneity: Chi² = 0.14, df = 1 (P = 0.71); I² = 0%						
Test for overall effect: Z = 2.32 (P = 0.02)						
**1.9.2 >4wk, ≤8wk**						
Huang 2016	0	38	0	40		Not estimable
Liu 2010	0	30	18	30	43.0%	0.03 [0.00, 0.43]
Zhang 2011	0	30	6	30	15.1%	0.08 [0.00, 1.31]
**Subtotal (95% CI)**		**98**		**100**	**58.1%**	**0.04 [0.01, 0.29]**
Total events	0		24			
Heterogeneity: Chi² = 0.28, df = 1 (P = 0.60); I² = 0%						
Test for overall effect: Z = 3.21 (P = 0.001)						
**1.9.3 >8wk**						
Huang 2014	2	20	7	20	16.3%	0.29 [0.07, 1.21]
**Subtotal (95% CI)**		**20**		**20**	**16.3%**	**0.29 [0.07, 1.21]**
Total events	2		7			
Heterogeneity: Not applicable						
Test for overall effect: Z = 1.70 (P = 0.09)						
**Total (95% CI)**		**207**		**209**	**100.0%**	**0.09 [0.03, 0.25]**
Total events	2		41			
Heterogeneity: Chi² = 3.24, df = 4 (P = 0.52); I² = 0%						
Test for overall effect: Z = 4.67 (P < 0.00001)						
Test for subgroup differences: Chi² = 2.64, df = 2 (P = 0.27), I² = 24.2%						

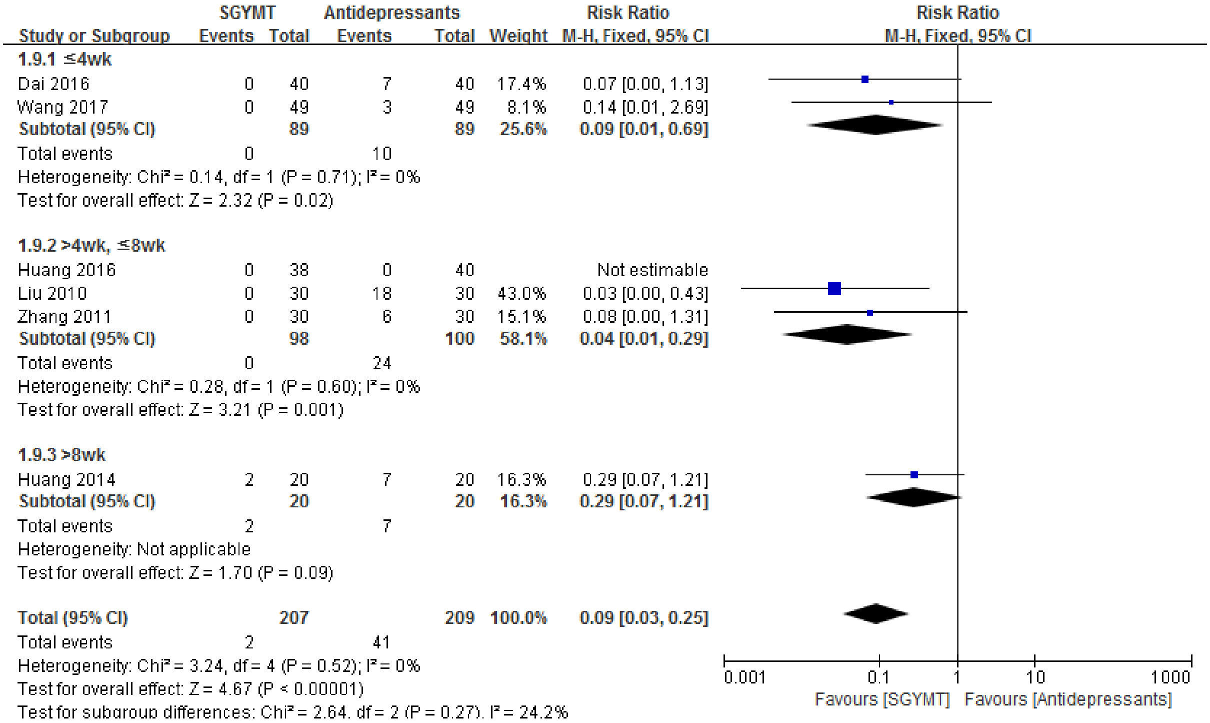

(h) Incidence of adverse events (SGYMT versus Antidepressants). Subgroup analysis according to dosage form

Study or Subgroup	SGYMT Events	SGYMT Total	Antidepressants Events	Antidepressants Total	Weight	Risk Ratio M-H, Fixed, 95% CI
**3.9.1 decoction**						
Huang 2014	2	20	7	20	16.3%	0.29 [0.07, 1.21]
Huang 2016	0	38	0	40		Not estimable
Liu 2010	0	30	18	30	43.0%	0.03 [0.00, 0.43]
Wang 2017	0	49	3	49	8.1%	0.14 [0.01, 2.69]
Zhang 2011	0	30	6	30	15.1%	0.08 [0.00, 1.31]
**Subtotal (95% CI)**		**167**		**169**	**82.6%**	**0.10 [0.03, 0.29]**
Total events	2		34			
Heterogeneity: Chi² = 3.02, df = 3 (P = 0.39); I² = 1%						
Test for overall effect: Z = 4.26 (P < 0.0001)						
**3.9.2 granule**						
Dai 2016	0	40	7	40	17.4%	0.07 [0.00, 1.13]
**Subtotal (95% CI)**		**40**		**40**	**17.4%**	**0.07 [0.00, 1.13]**
Total events	0		7			
Heterogeneity: Not applicable						
Test for overall effect: Z = 1.88 (P = 0.06)						
**Total (95% CI)**		**207**		**209**	**100.0%**	**0.09 [0.03, 0.25]**
Total events	2		41			
Heterogeneity: Chi² = 3.24, df = 4 (P = 0.52); I² = 0%						
Test for overall effect: Z = 4.67 (P < 0.00001)						
Test for subgroup differences: Chi² = 0.06, df = 1 (P = 0.80), I² = 0%						

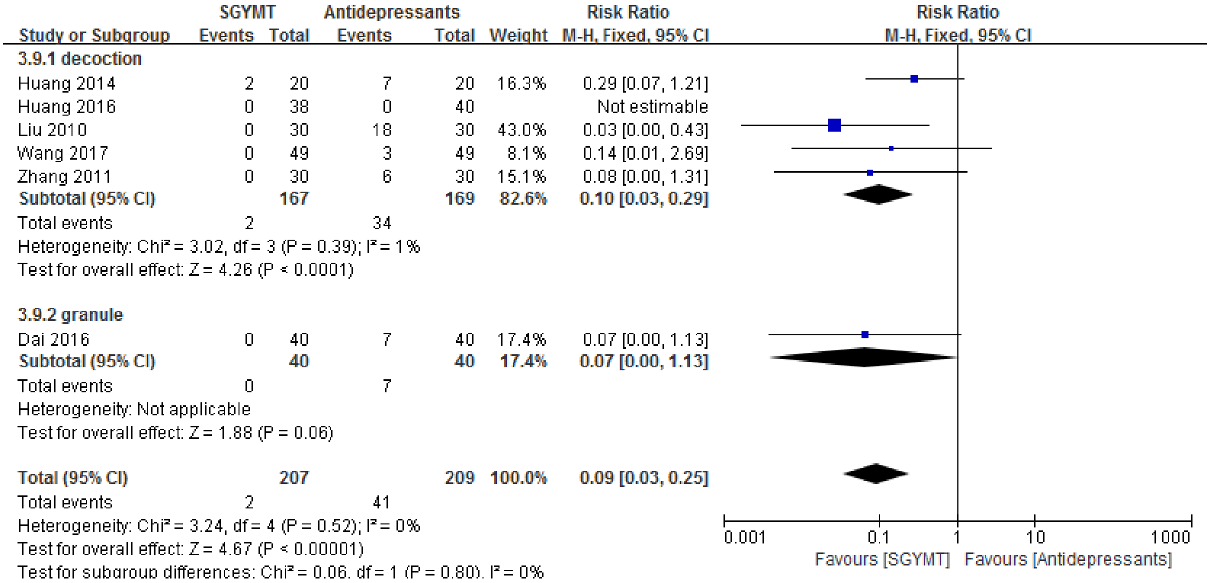

(i) Incidence of adverse events (SGYMT versus Antidepressants). Subgroup analysis according to kinds of antidepressant

Study or Subgroup	SGYMT Events	SGYMT Total	Antidepressants Events	Antidepressants Total	Weight	Risk Ratio M-H, Fixed, 95% CI
**7.9.1 SSRI**						
Huang 2014	2	20	7	20	16.3%	0.29 [0.07, 1.21]
Liu 2010	0	30	18	30	43.0%	0.03 [0.00, 0.43]
Wang 2017	0	49	3	49	8.1%	0.14 [0.01, 2.69]
**Subtotal (95% CI)**		**99**		**99**	**67.4%**	**0.10 [0.03, 0.33]**
Total events	2		28			
Heterogeneity: Chi² = 2.85, df = 2 (P = 0.24); I² = 30%						
Test for overall effect: Z = 3.87 (P = 0.0001)						
**7.9.2 Flupentixol/melitracen**						
Dai 2016	0	40	7	40	17.4%	0.07 [0.00, 1.13]
Huang 2016	0	38	0	40		Not estimable
Zhang 2011	0	30	6	30	15.1%	0.08 [0.00, 1.31]
**Subtotal (95% CI)**		**108**		**110**	**32.6%**	**0.07 [0.01, 0.53]**
Total events	0		13			
Heterogeneity: Chi² = 0.00, df = 1 (P = 0.94); I² = 0%						
Test for overall effect: Z = 2.58 (P = 0.010)						
**Total (95% CI)**		**207**		**209**	**100.0%**	**0.09 [0.03, 0.25]**
Total events	2		41			
Heterogeneity: Chi² = 3.24, df = 4 (P = 0.52); I² = 0%						
Test for overall effect: Z = 4.67 (P < 0.00001)						
Test for subgroup differences: Chi² = 0.10, df = 1 (P = 0.75), I² = 0%						

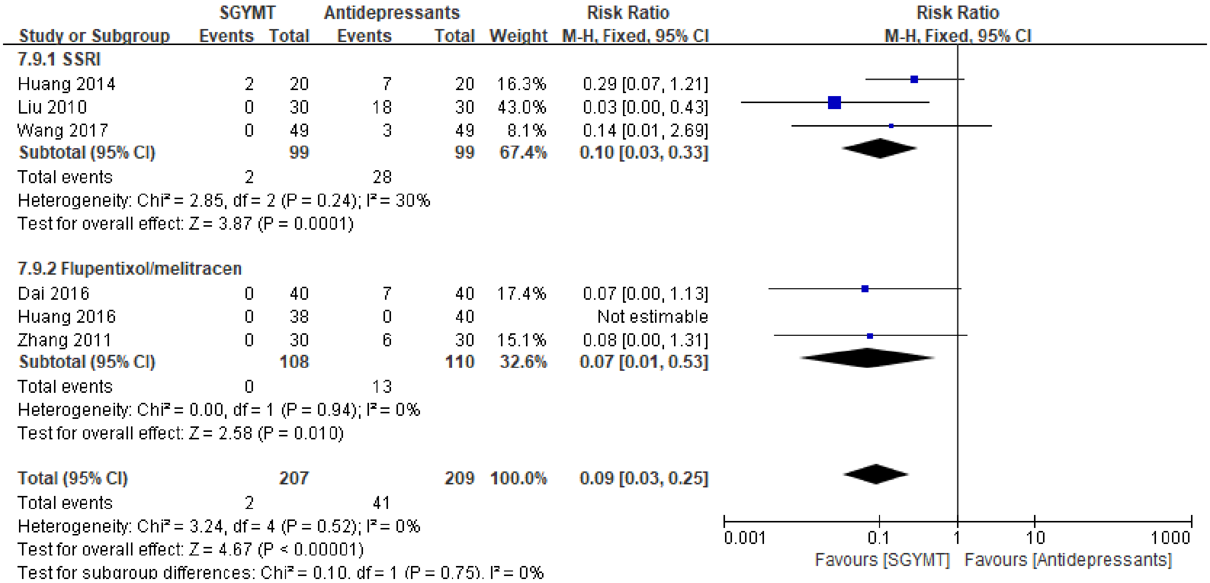

Supplemental Digital Content 8. PRISMA 2009 checklist.

PRISMA 2009 Checklist

Section/topic	#	Checklist item	Reported on page #
**TITLE**			
Title	1	Identify the report as a systematic review, meta-analysis, or both.	1
**ABSTRACT**			
Structured summary	2	Provide a structured summary including, as applicable: background; objectives; data sources; study eligibility criteria, participants, and interventions; study appraisal and synthesis methods; results; limitations; conclusions and implications of key findings; systematic review registration number.	2
**INTRODUCTION**			
Rationale	3	Describe the rationale for the review in the context of what is already known.	4-5
Objectives	4	Provide an explicit statement of questions being addressed with reference to participants, interventions, comparisons, outcomes, and study design (PICOS).	5
**METHODS**			
Protocol and registration	5	Indicate if a review protocol exists, if and where it can be accessed (e.g., Web address), and, if available, provide registration information including registration number.	6
Eligibility criteria	6	Specify study characteristics (e.g., PICOS, length of follow-up) and report characteristics (e.g., years considered, language, publication status) used as criteria for eligibility, giving rationale.	6-7
Information sources	7	Describe all information sources (e.g., databases with dates of coverage, contact with study authors to identify additional studies) in the search and date last searched.	6, 8
Search	8	Present full electronic search strategy for at least one database, including any limits used, such that it could be repeated.	S1
Study selection	9	State the process for selecting studies (i.e., screening, eligibility, included in systematic review, and, if applicable, included in the meta-analysis).	7
Data collection process	10	Describe method of data extraction from reports (e.g., piloted forms, independently, in duplicate) and any processes for obtaining and confirming data from investigators.	7-8
Data items	11	List and define all variables for which data were sought (e.g., PICOS, funding sources) and any assumptions and simplifications made.	6-8
Risk of bias in individual studies	12	Describe methods used for assessing risk of bias of individual studies (including specification of whether this was done at the study or outcome level), and how this information is to be used in any data synthesis.	8-9
Summary measures	13	State the principal summary measures (e.g., risk ratio, difference in means).	9
Synthesis of results	14	Describe the methods of handling data and combining results of studies, if done, including measures of consistency (e.g., I^2) for each meta-analysis.	9

PRISMA 2009 Checklist

Section/topic	#	Checklist item	Reported on page #
Risk of bias across studies	15	Specify any assessment of risk of bias that may affect the cumulative evidence (e.g., publication bias, selective reporting within studies).	9
Additional analyses	16	Describe methods of additional analyses (e.g., sensitivity or subgroup analyses, meta-regression), if done, indicating which were pre-specified.	9
**RESULTS**			
Study selection	17	Give numbers of studies screened, assessed for eligibility, and included in the review, with reasons for exclusions at each stage, ideally with a flow diagram.	10, Figure 1
Study characteristics	18	For each study, present characteristics for which data were extracted (e.g., study size, PICOS, follow-up period) and provide the citations.	10-11, Table 1, S3
Risk of bias within studies	19	Present data on risk of bias of each study and, if available, any outcome level assessment (see item 12).	10-11, S2 Figure 2, 3
Results of individual studies	20	For all outcomes considered (benefits or harms), present, for each study: (a) simple summary data for each intervention group (b) effect estimates and confidence intervals, ideally with a forest plot.	11-13 Figure 4-6, S5, 6
Synthesis of results	21	Present results of each meta-analysis done, including confidence intervals and measures of consistency.	11-13, Table 2, 3
Risk of bias across studies	22	Present results of any assessment of risk of bias across studies (see Item 15).	10-11, 13, Figure 7
Additional analysis	23	Give results of additional analyses, if done (e.g., sensitivity or subgroup analyses, meta-regression [see Item 16]).	11-13, S4 Table 2, 3
**DISCUSSION**			
Summary of evidence	24	Summarize the main findings including the strength of evidence for each main outcome; consider their relevance to key groups (e.g., healthcare providers, users, and policy makers).	14-15
Limitations	25	Discuss limitations at study and outcome level (e.g., risk of bias), and at review-level (e.g., incomplete retrieval of identified research, reporting bias).	15-16
Conclusions	26	Provide a general interpretation of the results in the context of other evidence, and implications for future research.	14-17
**FUNDING**			
Funding	27	Describe sources of funding for the systematic review and other support (e.g., supply of data); role of funders for the systematic review.	18

From: Moher D, Liberati A, Tetzlaff J, Altman DG, The PRISMA Group (2009). Preferred Reporting Items for Systematic Reviews and Meta-Analyses: The PRISMA Statement. PLoS Med 6(7): e1000097. doi:10.1371/journal.pmed1000097

For more information, visit: www.prisma-statement.org.
